# Trans-synaptic zinc mobilization improves social interaction in two mouse models of autism through NMDAR activation

**DOI:** 10.1038/ncomms8168

**Published:** 2015-05-18

**Authors:** Eun-Jae Lee, Hyejin Lee, Tzyy-Nan Huang, Changuk Chung, Wangyong Shin, Kyungdeok Kim, Jae-Young Koh, Yi-Ping Hsueh, Eunjoon Kim

**Affiliations:** 1 Graduate School of Medical Science and Engineering, Korea Advanced Institute of Science and Technology, Daejeon 305-701, Korea; 2 Center for Synaptic Brain Dysfunctions, Institute for Basic Science (IBS), Daejeon 305-701, Korea; 3 Department of Biological Sciences, Korea Advanced Institute of Science and Technology, Daejeon 305-701, Korea; 4 Institute of Molecular Biology, Academia Sinica, Taipei 115, Taiwan; 5 Neural Injury Research Lab, University of Ulsan College of Medicine, Seoul 138-736, Korea; 6 Asan Institute for Life Science, University of Ulsan College of Medicine, Seoul 138-736, Korea; 7 Department of Neurology, University of Ulsan College of Medicine, Seoul 138-736, Korea

## Abstract

Genetic aspects of autism spectrum disorders (ASDs) have recently been extensively explored, but environmental influences that affect ASDs have received considerably less attention. Zinc (Zn) is a nutritional factor implicated in ASDs, but evidence for a strong association and linking mechanism is largely lacking. Here we report that trans-synaptic Zn mobilization rapidly rescues social interaction in two independent mouse models of ASD. In mice lacking Shank2, an excitatory postsynaptic scaffolding protein, postsynaptic Zn elevation induced by clioquinol (a Zn chelator and ionophore) improves social interaction. Postsynaptic Zn is mainly derived from presynaptic pools and activates NMDA receptors (NMDARs) through postsynaptic activation of the tyrosine kinase Src. Clioquinol also improves social interaction in mice haploinsufficient for the transcription factor Tbr1, which accompanies NMDAR activation in the amygdala. These results suggest that trans-synaptic Zn mobilization induced by clioquinol rescues social deficits in mouse models of ASD through postsynaptic Src and NMDAR activation.

## Abstract (web summary)

Zinc is a nutritional factor implicated in autism spectrum disorders (ASDs), but evidence for a strong association and linking mechanism is largely lacking. Here, the authors report that trans-synaptic zinc mobilization rapidly rescues social interaction in two independent mouse models of ASD.

## Graphical abstract

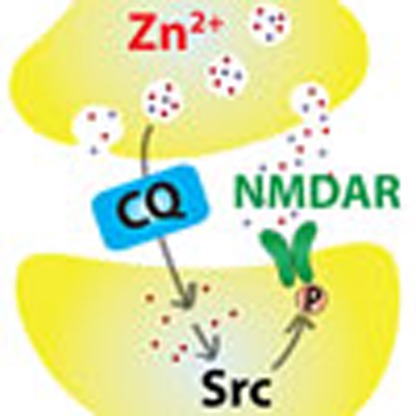

Autism spectrum disorders (ASDs) represent a neurodevelopmental disorder characterized by impaired social interaction and communication, and restricted and repetitive behaviour, interest and activity. ASDs affect ∼1% of the population and are thought to be strongly influenced by genetic factors. A large number of ASD-associated genetic variations have recently been identified, indicating that ASDs represent a genetically heterogeneous family of disorders^1^,^2^,^3^. Some of the genetic variations lie along common pathways/functions, including synaptic transmission, transcriptional regulation and chromatin remodelling^1^,^2^,^3^. In addition, studies using mouse models of ASD carrying these mutations have begun to suggest possible mechanisms that may underlie the pathogenesis of ASD, namely glutamatergic dysfunction and an imbalance between excitatory and inhibitory synapses^4^,^5^,^6^,^7^,^8^,^9^,^10^,^11^,^12^,^13^,^14^.

Environmental influences, such as nutrition, toxins and poisons, drugs, infection and stress, are thought to have a significant influence on psychiatric disorders. In ASDs, well-known examples of environmental influences include pre- or perinatal exposure to viruses or teratogens such as valproic acid and thalidomide^15^,^16^. However, studies on additional environmental influences and underlying mechanisms are at an early stage. This contrasts with the rapidly growing evidence for the contribution of genetic factors to ASDs. Because environmental factors are highly likely to interact with the genetic variations of ASD to determine the type, severity and trajectory of ASD symptoms, a balance between genetic and environmental causes is required in studies of ASDs.

Zinc (Zn), the second-most abundant trace element with a critical role in human nutrition and health, regulates a variety of cellular processes and protein functions. Zn deficiency has been implicated in diverse neurological and psychiatric disorders, including Alzheimer's disease, Parkinson's disease, ASDs, attention deficit/hyperactivity disorder, schizophrenia, epilepsy and mood disorders^17^. The association of Zn with ASDs has been suggested based on its deficiency in individuals with ASDs, including a recent large cohort of 1,967 children^16^,^18^, as well as the phenotypes of Zn-deficient experimental animals^19^. This association is further supported by the potential therapeutic value of Zn supplementation in ASD treatment^17^,^20^. However, strong evidence supporting the association between Zn deficiency and ASDs is largely unavailable, and the mechanisms underlying the association remain obscure.

In the synapse, the main pool of Zn ions is presynaptic vesicles where Zn is in the millimolar range, whereas postsynaptic sites contain much smaller amounts of Zn (picomolar range)^21^,^22^,^23^,^24^. Presynaptic free Zn is co-released with glutamate during neuronal activity and serves to suppress NMDA receptors (NMDARs) in the synaptic cleft. Some Zn ions enter the postsynaptic sites through calcium channels, NMDARs and calcium-permeable AMPA receptors (AMPARs), and regulate target proteins such as NMDARs and TrkB receptors through mechanisms including those involving Src family tyrosine kinases (SFKs)^25^,^26^,^27^. Another important effector of postsynaptic Zn is Shank (also known as ProSAP), a family of excitatory postsynaptic scaffolding proteins with three known members (Shank1/2/3; refs 28, 29). Zn binds to Shank2/3 and enhances their postsynaptic stabilization, promoting excitatory synapse formation and maturation^30^.

Shank2/3, members of the Shank family of postsynaptic scaffolding proteins (also known as ProSAP1/2), have been implicated in ASDs through human genetic studies^31^,^32^,^33^,^34^,^35^,^36^ and mouse model/cultured neuron studies^19^,^30^,^37^,^38^,^39^,^40^,^41^,^42^,^43^,^44^,^45^,^46^,^47^,^48^. Mice carrying Shank2/3 mutations display diverse dysfunctions at glutamate synapses^40^,^41^,^42^,^43^,^44^,^45^,^46^,^49^. One notable change is the reduction in NMDAR function observed in *Shank2*^*−/−*^ mice (exons 6+7 deletion)^45^. In these mice, normalization of NMDAR function with an NMDAR agonist (D-cycloserine) is associated with the rescue of impaired social interaction, suggesting that NMDAR hypofunction might underlie the social deficit in these mice. Although validation of this hypothesis will require further analyses, D-cycloserine has also been shown to rescue the impaired social interaction in mice with a haploinsufficiency of the transcription factor Tbr1 (T-box brain 1; ref. 50), which positively regulates the expression of *Grin2b* (ref. 51), encoding the GluN2B subunit of NMDARs.

In the present study, we demonstrate that trans-synaptic Zn mobilization by clioquinol, a Zn chelator and ionophore (termed CQ hereafter), rescues the social interaction deficits in *Shank2*^*−/−*^ and *Tbr1*^*+/−*^ mice. CQ mobilizes Zn from enriched presynaptic pools to postsynaptic sites, where it enhances NMDAR function through Src activation. These results indicate that postsynaptic Zn rescues social interaction deficits in distinct mouse models of ASDs, and suggest that reduced NMDAR function is associated with ASDs.

## Results

### CQ rapidly improves social interaction in *Shank2* ^*−/−*^ mice

Based on the close associations among Zn, NMDAR, Shank and ASD mentioned above, we reasoned that Zn delivered to postsynaptic compartments might rescue the reduced NMDAR function and ASD-like behaviours observed in *Shank2*^*−/−*^ mice. To test this idea, we first intraperitoneally (i.p.) injected *Shank2*^*−/−*^ mice with CQ (30 mg kg^−1^), a lipophilic Zn chelator (*K*_d_≈10^−7^) and ionophore that readily crosses the blood–brain barrier and mobilizes Zn down a concentration gradient^52^. We chose systemic administration of CQ because dietary Zn is known to have poor bioavailability and side effects including gastric irritation^17^,^53^.

**Figure 1:**
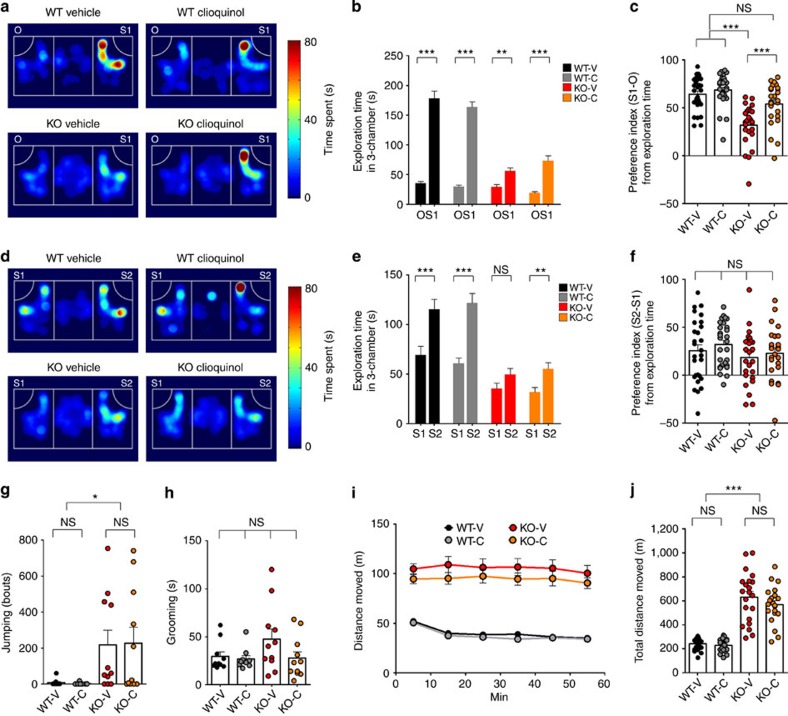
CQ treatment rapidly improves social interaction in *Shank2*^*−/−*^ mice. (**a**–**f**) CQ improves social interaction in *Shank2*^*−/−*^ (KO) mice, but has no effect on WT mice (**a**–**c**). Note that levels of social novelty recognition are similar in WT and *Shank2*^*−/−*^ mice, and that CQ does not affect social novelty recognition in these mice (**d**–**f**). Mice were injected with CQ (30 mg kg^−1^; i.p.), or vehicle, 2 h before behavioural tests. Heat maps in (**a**,**d**) represent examples of mouse movements. The social preference index from exploration time represents the numerical difference between the times spent exploring or sniffing the two targets (S1/stranger versus O/object or S2/new stranger versus S1/previous stranger) divided by total time spent × 100. V, vehicle; C, CQ. (*n*=28 for WT-V and WT-C, and 25 for KO-V and KO-C); **P<0.01, ***P<0.001; Kruskal–Wallis one-way analysis of variance (ANOVA) with Dunn's *post hoc* test). (**g**,**h**) CQ has no effect on jumping or grooming behaviour in *Shank2*^*−/−*^ mice. (*n*=10 for WT-V and WT-C, and 11 for KO-V and KO-C, *P<0.05, two-way ANOVA and Kruskal–Wallis one-way ANOVA with Dunn's *post hoc* test). (**i**,**j**) CQ does not normalize hyperactivity in *Shank2*^*−/−*^ mice. (*n*=23 for WT-V and WT-C, and 21 for KO-V and KO-C; ***P<0.001, two-way ANOVA and Kruskal–Wallis one-way ANOVA with Dunn's *post hoc* test). Data in all panels with error bars represent mean±s.e.m. NS, not significant.

Two hours after CQ treatment, the mice were subjected to the three-chamber social interaction test, which compares the preference of a mouse for a stranger mouse versus a novel inanimate object. We found that *Shank2*^*−/−*^ mice displayed reduced social interaction compared with wild-type (WT) mice, as determined by time spent exploring/sniffing the target and the social preference indices derived from exploration time (see figure legend for details) (Fig. 1a–c and Supplementary Fig. 1a,d–i), consistent with previous results from untreated *Shank2*^*−/−*^ and WT mice^45^. This impairment was improved by CQ treatment. In contrast, social interaction in WT mice was not affected by CQ. Notably, *Shank2*^*−/−*^ mice in the chamber with a stranger often spent time in other activities such as jumping, contrary to WT mice, as reflected in the relatively low correlation between exploration time and chamber time (Supplementary Fig. 2), and showed an apparent lack of CQ-dependent improvement in social interaction, as determined by the time spent in chamber and the preference index derived from chamber time (Supplementary Fig. 1b,c).

When social novelty recognition was determined in the same three-chamber test, by measuring the preference for a previously encountered stranger mouse versus a new stranger mouse, *Shank2*^*−/−*^ mice showed levels of social novelty recognition comparable to those in WT mice (Fig. 1d–f and Supplementary Fig. 1j–m), as reported previously^45^. In addition, CQ had no effect on social novelty recognition in both WT and *Shank2*^*−/−*^ mice.

The CQ-dependent rescue of social interaction in *Shank2*^*−/−*^ mice but no effect of CQ on WT mice is unlikely attributable to differences in the amount of free Zn available for CQ binding in these mice, because total levels of free Zn measured with the fluorescent dye TFL-Zn were not different (Supplementary Fig. 3a,b). In addition, the levels of Zn transporter 3 (ZnT3), a protein required for Zn transport into presynaptic vesicles^54^, which is the main pool of free Zn in the brain, were similar between genotypes (Supplementary Fig. 3c). Finally, whole-brain levels of Zn, Cu or Fe were not different between WT and *Shank2*^*−/−*^ mice, which is in line with the above-mentioned TFL-Zn staining result, and, more importantly, 2-hr CQ treatment did not cause an acute reduction in the levels of these metals in WT or *Shank2*^*−/−*^ mice (Supplementary Fig. 4a–c), suggesting that the chelating activity of CQ unlikely contributes to the observed social rescue.

In repetitive behaviour assays, vehicle-treated *Shank2*^*−/−*^ mice showed increased jumping behaviour but normal grooming in their home cages, relative to vehicle-treated WT mice, consistent with the previous results^45^; these behaviours were unaffected by CQ (Fig. 1g,h and Supplementary Fig. 5a–d). Similarly, CQ did not affect repetitive behaviours in WT mice.

In the open-field test, *Shank2*^*−/−*^ mice displayed increased locomotor activity relative to WT mice, as previously reported. This hyperactivity was not attenuated by CQ (Fig. 1i,j). Notably, *Shank2*^*−/−*^ mice spent less time in the centre region of the open-field arena, a measure of anxiety-like behaviour. However, CQ had no effect on the centre-region time in these mice (Supplementary Fig. 5e). CQ did not affect the repetitive behaviour, locomotor activity or anxiety-like behaviour of WT mice. Together, these results suggest that CQ improves social interaction but has no effect on social novelty recognition, repetitive behaviour, hyperactivity or anxiety-like behaviour in *Shank2*^*−/−*^ mice.

### NMDAR function at *Shank2* ^*−/−*^ synapses is restored by CQ

**Figure 2:**
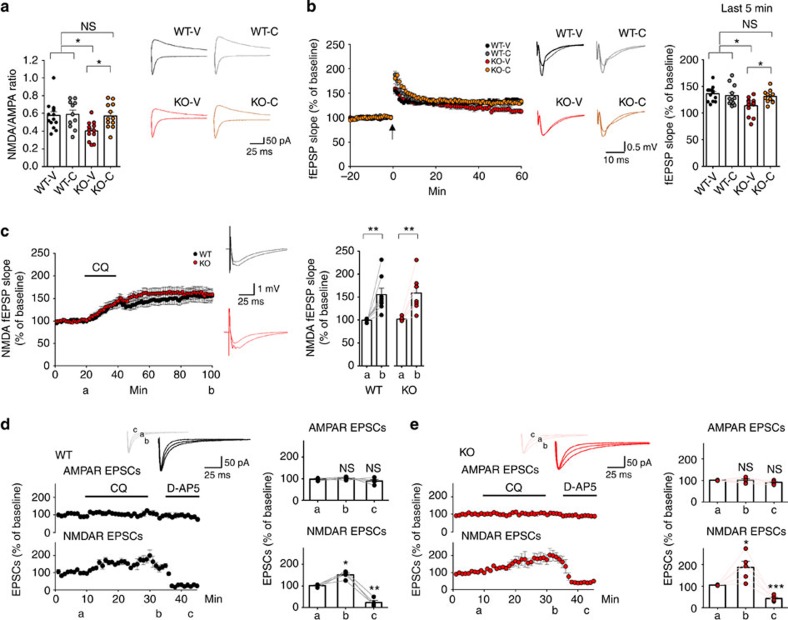
CQ restores NMDAR function at ***Shank2***^***−/−***^ **synapses.** (**a**) CQ (4 μM) normalizes the NMDA/AMPA ratio at *Shank2*^*−/−*^ hippocampal SC-CA1 synapses (P17–25), as measured by NMDA- and AMPA-eEPSCs. Representative eEPSC traces recorded at −70 and+40 mV. NMDA-eEPSCs were measured at +40 mV holding potential, 60 ms after the stimulation. (*n*=14 cells (from 10 animals) for WT-V, 11 (7) for WT-C, 12 (8) for KO-V and 12 (7) for KO-C, *P<0.05; one-way analysis of variance (ANOVA) with Tukey's *post hoc* test). (**b**) CQ (4 μM) restores LTP, induced by tetanus (100 Hz), at *Shank2*^*−/−*^ hippocampal SC-CA1 synapses (3–5 weeks), as measured by fEPSPs. (*n*=13 slices (from 8 animals) for WT-V, 12 (5) for WT-C, 13 (7) for KO-V and 11 (5) for KO-C; *P<0.05; one-way ANOVA with Tukey's *post hoc* test). (**c**) CQ (4 μM, 20 min) enhances NMDAR function at WT and *Shank2*^*−/−*^ hippocampal SC-CA1 synapses, as measured by NMDA-fEPSPs. (*n*=8 slices (from 7 animals) for WT and 8 (7) for KO; **P<0.01; Student's *t*-test) The labels a and b indicate 5-min duration before CQ and the end of recording, respectively. (**d**,**e**) CQ (4 μM, 20 min) enhances NMDAR function at WT and *Shank2*^*−/−*^ hippocampal SC-CA1 synapses, as determined by simultaneous measurements of NMDA- and AMPA-eEPSCs at −40 mV. NMDA-eEPSCs were measured at 60 ms after stimulation. D-AP5 (50 μM, 10 min) was used to test NMDAR dependence. The labels (**a**–**c**) indicate 5-min duration before and after CQ, and at the end of recording, respectively. (*n*=4 cells (three slices) for WT and 5 cells (four slices) for KO; *P<0.05, **P<0.01, ***P<0.001; Student's *t*-test, compared with the 5-min duration before CQ). Data in all panels with error bars represent mean±s.e.m. NS, not significant.

The CQ-dependent rescue of social interaction deficits in *Shank2*^*−/−*^ mice could involve normalization of the reported reduction in NMDAR function in these mice^45^. Consistent with this possibility, we found that CQ treatment restored normal levels of NMDAR function at *Shank2*^*−/−*^ hippocampal Schaffer collateral-CA1 pyramidal (SC-CA1) synapses, as determined by the ratio of NMDAR- to AMPAR-evoked excitatory postsynaptic currents (NMDA/AMPA ratio of eEPSCs; Fig. 2a). CQ, however, had no effect at WT synapses. In addition, CQ reversed the reduced tetanus-induced long-term potentiation (LTP), known to require NMDAR activity, at *Shank2*^*−/−*^ SC-CA1 synapses, but had no effect on LTP at WT synapses (Fig. 2b). These results indicate that CQ restores NMDAR function at *Shank2*^*−/−*^ hippocampal SC-CA1 synapses.

We next measured the time course of CQ-dependent NMDAR activation at *Shank2*^*−/−*^ synapses. In these experiments, we treated *Shank2*^*−/−*^ hippocampal slices with CQ for 20 min (in contrast to the continuous bath application for experiments described above) while monitoring NMDAR-mediated field excitatory postsynaptic potentials (NMDA-fEPSPs) before, during and after CQ treatment. With CQ treatment, the initial slopes of NMDA-fEPSPs at *Shank2*^*−/−*^ SC-CA1 synapses gradually increased to ∼150% of baseline levels and remained elevated, a response similar to that observed in WT slices (Fig. 2c). In contrast to NMDA-fEPSPs, AMPAR-mediated fEPSPs (AMPA-fEPSPs) were not affected by CQ treatment (Supplementary Fig. 6a). CQ also had no effect on AMPAR-related input–output ratio (AMPA-fEPSP slopes plotted against fibre volleys) or paired-pulse ratio at SC-CA1 synapses (Supplementary Fig. 6b,c).

To further confirm the CQ-dependent NMDAR activation, we simultaneously measured NMDA- and AMPA-eEPSCs using patch-clamp recordings. At a holding potential of −40 mV, CQ increased NMDA-eEPSCs at *Shank2*^*−/−*^ SC-CA1 synapses, a result similar to that observed at WT synapses (Fig. 2d,e). The NMDAR antagonist D-AP5 significantly reduced NMDA-eEPSCs but not AMPA-eEPSCs, indicating that these events are NMDAR dependent. In contrast, AMPA-eEPSCs were not affected by CQ treatment (Fig. 2d,e). Consistent with this, the NMDA/AMPA ratios derived from these currents were increased in both genotypes (Supplementary Fig. 6d,e). Taken together, these results indicate that CQ enhances NMDAR but not AMPAR function at *Shank2*^*−/−*^ and WT synapses.

### CQ mobilizes Zn from pre- to postsynaptic sites

**Figure 3:**
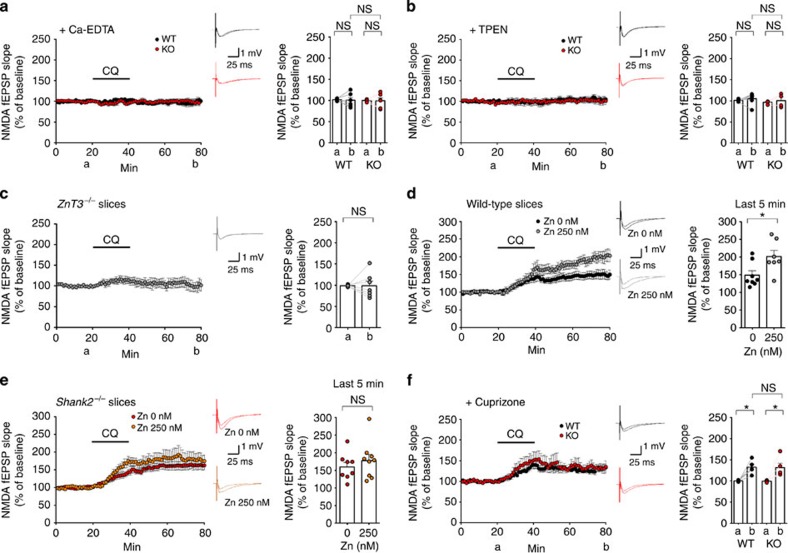
CQ-dependent NMDAR activation requires Zn mobilization from pre- to postsynaptic sites. (**a**,**b**) CQ (4 μM, 20 min) fails to enhance NMDAR function at hippocampal SC-CA1 synapses in the presence of Ca-EDTA or TPEN (Zn chelators more potent than CQ), as measured by NMDA-fEPSPs. *Shank2*^*−/−*^ hippocampal slices were bath incubated with Ca-EDTA (2 μM) or TPEN (25 μM) throughout recordings. The labels a and b indicate 5-min durations before CQ and the end of recordings, respectively. (Ca-EDTA, *n*=6 slices (3 animals) for WT and 6 (3) for KO; Student's *t*-test; TPEN, *n*=6 (2) for WT and 4 (2) for KO; Student's *t*-test). (**c**) CQ fails to enhance NMDAR function at *ZnT3*^*−/−*^ hippocampal SC-CA1 synapses. (*n*=7 slices (3 animals); Student's *t*-test). (**d**,**e**) Exogenously added Zn (250 nM) enhances NMDAR function at WT but not *Shank2*^*−/−*^ synapses. Additional Zn was bath applied throughout the recording. (*n*=8 slices (7 animals) for 0 nM, 7 (4) for 250 nM in WT, and 8 (5) for 0 nM, 9 (4) for 250 nM in KO; *P<0.05; Student's *t*-test). (**f**) CQ enhances NMDAR function in the presence of cuprizone (100 μM), a Cu^2+^-specific chelator. (*n*=5 slices (4 animals) for WT and 5 (4) for KO; *P<0.05; Student's *t*-test). Data in all panels with error bars represent mean±s.e.m. NS, not significant.

Next, we determined whether CQ-dependent NMDAR activation requires Zn. To test this, we used two different Zn chelators with much higher affinities for Zn than CQ: Ca-EDTA (*K*_d_≈10^−13^), which is membrane impermeable, and TPEN (*K*_d_≈10^−15^), which is membrane permeable. Preincubation of slices with Ca-EDTA before CQ treatment eliminated the CQ-dependent increase in NMDAR activity at *Shank2*^*−/−*^ SC-CA1 synapses, as measured by NMDA-fEPSPs (Fig. 3a). In a control experiment, Ca-EDTA by itself had no effect on NMDA-fEPSPs (Supplementary Fig. 7a), as reported previously^24^,^55^. TPEN also blocked CQ-dependent NMDAR activation (Fig. 3b), although TPEN by itself caused a small increase in the basal activity of NMDARs (Supplementary Fig. 7b). Collectively, these findings suggest that CQ requires Zn for NMDAR activation. In addition, the absence of an effect of Ca-EDTA alone on NMDA-fEPSPs suggests that CQ-dependent NMDAR activation is unlikely the result of disinhibition of NMDARs by CQ-mediated chelation of Zn in the synaptic cleft.

If the Zn-ionophoric activity of CQ is the more likely candidate mediator of NMDAR activation, this raises the question: what is the source of Zn for NMDAR activation? One possible candidate is the Zn pool in presynaptic neurotransmitter vesicles, a major source of Zn in the brain that requires the ZnT3 transporter for its maintenance^21^,^22^,^23^,^24^. In tests of this possibility using ZnT3-deficient (*ZnT3*^*−/−*^) mice, we found that CQ had no effect on NMDAR activity in *ZnT3*^*−/−*^ SC-CA1 synapses (Fig. 3c), suggesting that the presynaptic Zn pool is required for the CQ effect. In a control experiment, we confirmed that Zn signals are indeed largely absent in the *ZnT3*^*−/−*^ hippocampus (Supplementary Fig. 3d).

The results described to this point were obtained in experiments performed without exogenous addition of Zn to brain slices, relying on the physiological concentrations of free Zn in the extracellular space. Previous studies have reported free Zn concentrations in the central nervous system of ∼20 nM (ref. 56), although higher concentrations might be possible^24^. Here, we tested whether increasing the extracellular Zn concentration to 250 nM affected CQ-dependent NMDAR activation. We found that 250 nM Zn had no significant effect on CQ-dependent NMDAR activation at *Shank2*^*−/−*^ SC-CA1 synapses, as measured by NMDA-fEPSPs (Fig. 3d,e). This result suggests that additional Zn is not required for CQ-dependent NMDAR activation at *Shank2*^*−/−*^ synapses, implying that the presynaptic Zn pool under physiological conditions is sufficient for CQ-dependent NMDAR activation. Interestingly, 250 nM Zn caused a significant increase in NMDA-fEPSPs at WT synapses (Fig. 3d,e), a differential effect that warrants further investigation.

**Figure 4:**
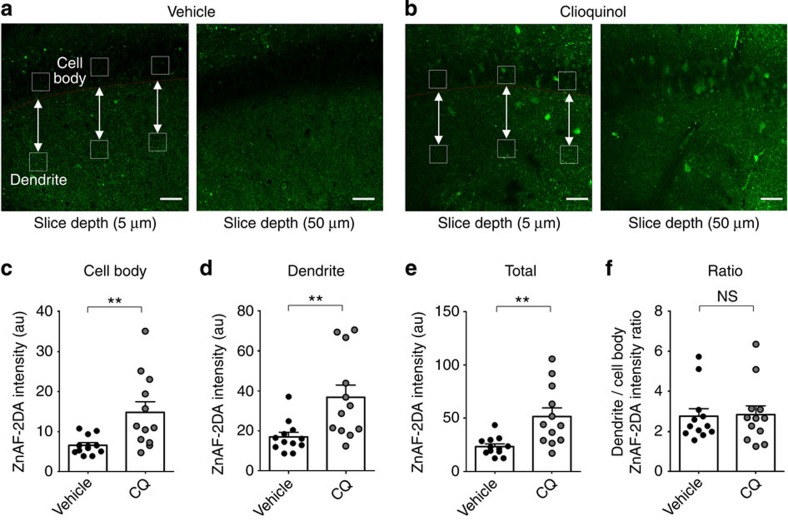
CQ treatment increases Zn signals in the dendritic and cell body areas in the hippocampal CA1 region. (**a**–**f**) Mice (3–5 weeks) were injected with CQ (30 mg kg^−1^; i.p), or vehicle, 2 h before brain slicing, and hippocampal slices (300 μm thick) were incubated with ZnAF-2DA (40 min) followed by two-photon microscopy. Zn signals (average intensities) were measured in the indicated three square regions in the cell body, or dendritic, area of the hippocampal CA1 region. Scale bars, 50 μm (**a**,**b**). (*n*=12 slices (6 animals) for vehicle and 12 slices (5 animals) for CQ; *P<0.05, **P<0.01, Student's *t*-test). Data in all panels with error bars represent mean±s.e.m. Au, arbitrary unit; NS, not significant.

We next attempted to visualize CQ-dependent increases in Zn levels in postsynaptic compartments, using ZnAF-2DA, a membrane-permeable Zn indicator that, once inside the cell, is modified and trapped to indicate intracellular Zn levels^57^,^58^. When WT mice were treated with CQ for 2 h, Zn signals measured by two-photon confocal microscopy were significantly increased in both dendritic and cell body area of the hippocampal CA1 region, compared with vehicle-treated controls (Fig. 4a–f; and Supplementary Movies 1 and 2), consistent with the previous results obtained using a regular confocal microscope^58^.

Finally, because CQ can bind Cu^2+^ (*K*_d_≈10^−8.9^) in addition to Zn, we tested whether the Cu^2+^-binding activity of CQ also contributed to its effects on NMDAR function. Application of cuprizone, a selective Cu^2+^ chelator, to hippocampal slices before CQ treatment did not inhibit the CQ-induced increase in NMDAR activity (Fig. 3f), suggesting that the Cu-binding activity of CQ is not important for NMDAR activation. Taken together, these results suggest that CQ enhances NMDAR function through its Zn-ionophoric, but not Zn-chelating or Cu^2+^-binding, activity, and uses mainly the presynaptic Zn pool for NMDAR activation.

### CQ activates NMDARs through postsynaptic Src

The results described thus far suggest that CQ mobilizes Zn from presynaptic vesicles into the synaptic cleft and postsynaptic compartments. Zn in the synaptic cleft is unlikely to contribute to NMDAR activation because Zn released presynaptically during neuronal activity is known to inhibit NMDARs^24^, and we demonstrated that Ca-EDTA has no effect on NMDA-fEPSPs (Supplementary Fig. 7a).

**Figure 5:**
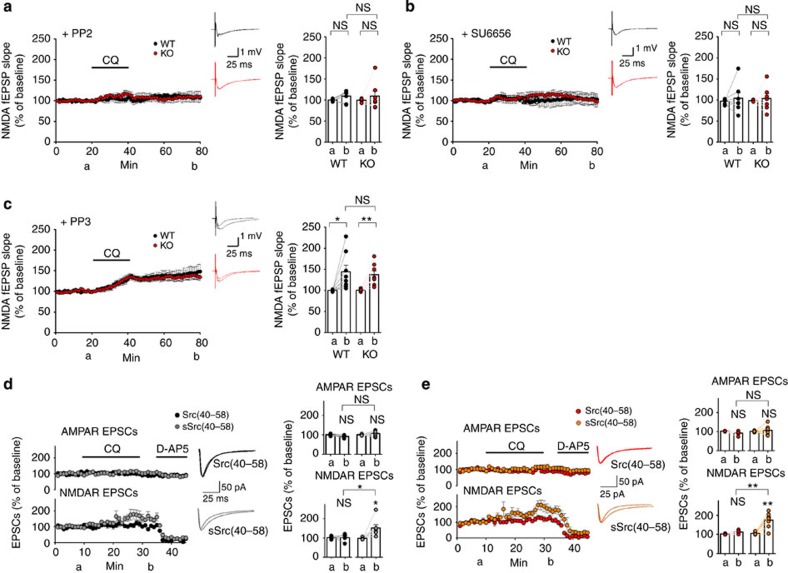
CQ treatment activates NMDARs through postsynaptic Src activation. (**a**–**c**) CQ (4 μM, 20 min) fails to enhance NMDAR function at *Shank2*^*−/−*^ SC-CA1 synapses in the presence of PP2 or SU6656, specific inhibitors of SFKs, but effectively enhances NMDAR function in the presence of PP3, an inactive PP2 analogue, as measured by NMDA-fEPSPs. Hippocampal slices were bath incubated with PP2 (10 μM) or SU6656 (10 μM) throughout recordings. (PP2, *n*=7 slices (6 animals) for WT and 7 (5) for KO; SU6656, *n*=7 (5) for WT and 7 (4) for KO; PP3, *n*=8 (4) for WT and 8 (5) for KO; *P<0.05, **P<0.01; Student's *t*-test). (**d**,**e**) CQ fails to enhance NMDAR function at *Shank2*^*−/−*^ SC-CA1 synapses (**e**) in the presence of Src(40–58), a specific peptide inhibitor of Src, but effectively enhances NMDAR function in the presence of sSrc(40–58), a scrambled version of the peptide, as determined by simultaneous measurements of NMDA- and AMPA-eEPSCs at −40 mV. NMDA-eEPSCs were measured at 60 ms after the stimulation. The labels a and b indicate 5-min duration before and after CQ, respectively. (Src(40–58), *n*=5 cells (4 animals) for WT and 6 (4) for KO; sSrc(40–58), *n*=7 (5) for WT, 7 (6) for KO; *P<0.05, **P<0.01; Student's *t*-test, compared with the 5-min duration before CQ). Data in all panels with error bars represent mean±s.e.m. NS, not significant.

If postsynaptic Zn delivery is an important factor, then what would be the underlying mechanism for NMDAR activation? Previous studies have shown that Zn binds to and inactivates C-terminal Src kinase, a negative regulator of SFKs, which phosphorylate and activate NMDARs^27^,^59^. In related experiments, we found that two independent SFK inhibitors, PP2 and SU6656, applied to hippocampal slices before CQ treatment abolished CQ activation of NMDARs at both *Shank2*^*−/−*^ and WT SC-CA1 synapses (Fig. 5a,b). In control experiments, PP3, an inactive PP2 analogue, failed to block the NMDAR activation (Fig. 5c).

To further confirm that CQ enhances NMDAR function through SFKs and to test that the subcellular site of SFK action is indeed postsynaptic, we used the Src-inhibitory peptide Src(40–58), which contains a sequence corresponding to Src amino-acid residues 40–58 and selectively blocks endogenous Src but not other SFK members^59^,^60^,^61^. Inclusion of this peptide (0.03 mg ml^−1^) in the patch pipette during patch-clamp recordings prevented CQ from increasing NMDAR activity at *Shank2*^*−/−*^ and WT synapses, determined by measuring NMDA-eEPSCs (Fig. 5d,e). In control experiments, a scrambled Src-peptide variant (sSrc 40–58) had no effect on the CQ-dependent increase in NMDAR activity.

In contrast to NMDARs, AMPAR function was unaffected by the Src-inhibitory peptide, as determined from simultaneous recordings of AMPA-eEPSCs at both WT and *Shank2*^*−/−*^ synapses (Fig. 5d,e). Consistent with this, the CQ-dependent increase in the NMDA/AMPA ratio determined from these currents was blocked by the Src-inhibitory peptide, but not by the scrambled peptide (Supplementary Fig. 8).

**Figure 6:**
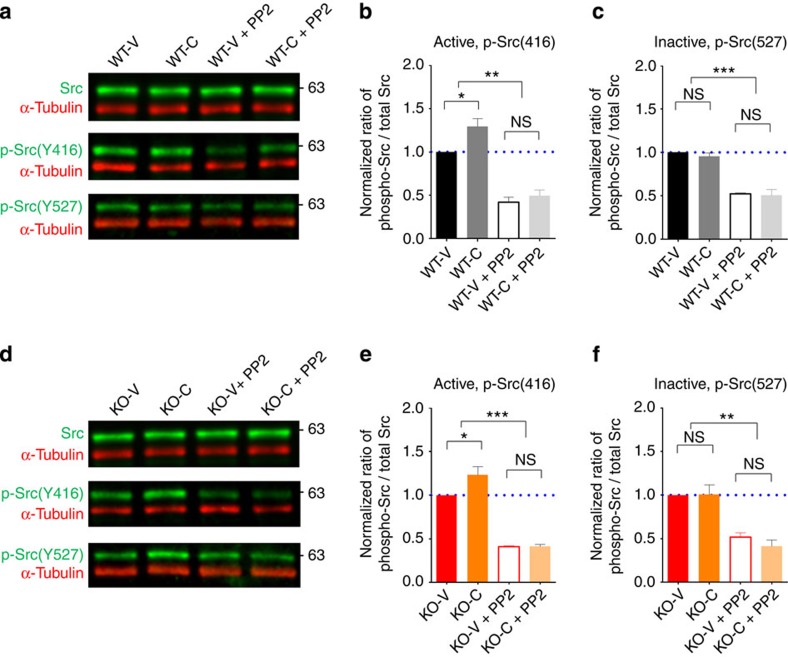
CQ increases Src tyrosine phosphorylation in the hippocampus. (**a**–**f**) Treatment of WT (**a**–**c**) and *Shank2*^*−/−*^ (**d**–**f**) hippocampal slices (3–5 weeks) with CQ in ACSF supplemented with 250 nM Zn for 20 min in the presence or absence of PP2 (SFK inhibitor) were followed by immunoblot analysis of total and tyrosine phosphorylated Src (Y416 and Y527). For quantification, levels of Src tyrosine phosphorylation were normalized to total Src levels. 250 nM Zn was added to maximize the visualization of the changes occurring in Src tyrosine phosphorylation. (*n*=6 slices (3 mice) for WT-V, WT-C, KO-V and KO-C; *P<0.05, **P<0.01, ***P<0.001; one-way analysis of variance with Tukey's *post hoc* analysis). Data in all panels with error bars represent mean±s.e.m. NS, not significant.

We also tested whether CQ treatment enhances Src activity, in addition to NMDAR function, by immunoblot analysis of CQ-treated hippocampal slices. We found that the levels of tyrosine phosphorylation of Src at Y416, known to render Src fully active^59^, were increased in CQ-treated WT and *Shank2*^*−/−*^ slices, which was blocked by PP2 (Fig. 6a,b,d,e). In contrast, tyrosine phosphorylation of Src at Y527, known to stabilize the inactive conformation of Src^59^, was not affected by CQ treatment (Fig. 6a,c,d,f), suggesting that CQ promotes Src activation through the phosphorylation of distinct tyrosine residues.

Finally, in order to explore the involvement of other signalling pathways in the downstream of NMDAR activation, we tested inhibitors of MAPK kinase/MEK (PD98059) and CaMKIIα (KN93). We found that suppression of MAPK/Erk by the inhibition of MAPKK/MEK in the upstream had no effect on CQ-dependent NMDAR activation (Supplementary Fig. 9a). Intriguingly, CaMKIIα inhibition caused a small reduction in the levels of CQ-induced NMDAR activation after but not during CQ treatment (Supplementary Fig. 9b), suggesting that CaMKIIα is required for the maintenance of enhanced NMDAR function. These results collectively suggest that the CQ-induced increase in NMDAR function is dependent on Src, and imply that the subcellular site of Src activation is postsynaptic.

### CQ treatment improves social interaction in *Tbr1* ^*+/−*^ mice

Finally, we considered whether CQ could rescue social interaction deficits in other mouse models of ASD in which reduced NMDAR is associated with autistic-like behaviours. One such model is the recently developed Tbr1-haploinsufficient (*Tbr1*^*+/−*^) mouse, which has been reported to display a reduction in social interaction that is normalized by the NMDAR agonist D-cycloserine^50^. We thus tested whether CQ could also rescue social interaction in these mice.

**Figure 7:**
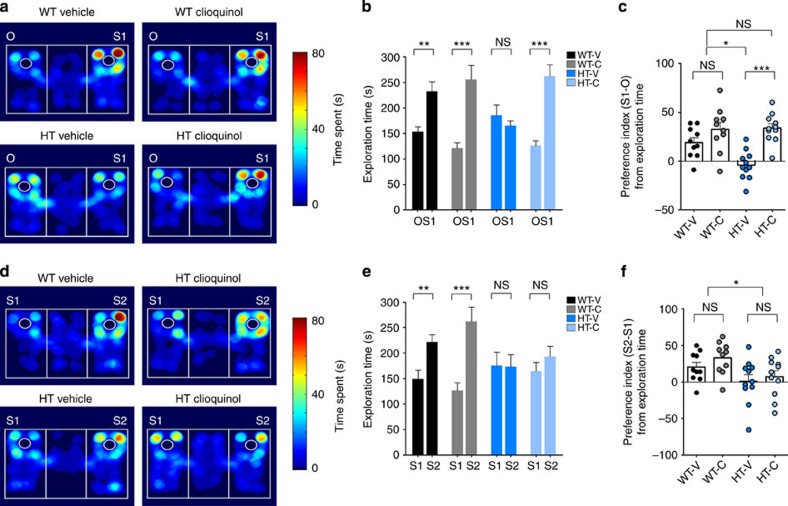
CQ treatment improves social interaction in *Tbr1*^*+/−*^ mice. (**a**–**f**) CQ improves social interaction (**a**–**c**) but not social novelty recognition (**d**–**f**) in *Tbr1*^*+/−*^ (HT) mice, but has no effect in WT mice, as measured by the time spent in exploring targets and the social preference index derived from these results. Heat maps in **a** and **d** represent examples of mouse movements. (*n*=10 for WT-V and WT-C, and 11 for HT-V and HT-C; *P<0.05, **P<0.01, ***P<0.001; two-way analysis of variance (ANOVA) and one-way ANOVA with Tukey's *post hoc* test). Data in all panels with error bars represent mean±s.e.m. NS, not significant.

*Tbr1*^*+/−*^ mice showed reduced social interaction in the three-chamber test, compared with WT mice, and acutely injected CQ (30 mg kg^−1^), administered 3 h before the three-chamber test, rescued the social interaction deficits of *Tbr1*^*+/−*^ mice, with no effect on WT mice, as determined by time spent in exploration/sniffing, time spent in chamber and the preference index derived from exploration or chamber time (Fig. 7a–c and Supplementary Fig. 10a–d). The positive rescue based on chamber time is supported by the strong correlation between exploration time and chamber time observed in *Tbr1*^*+/−*^ mice (Supplementary Fig. 11). CQ, however, did not affect social novelty recognition in *Tbr1*^*+/−*^ or WT mice, determined based on the preference for a new stranger mouse relative to a previously encountered stranger mouse (Fig. 7d–f and Supplementary Fig. 10e–h). These results suggest that CQ rescues social interaction deficits, but has no effect on social novelty recognition, in *Tbr1*^*+/−*^ mice. Taken together with similar results obtained in *Shank2*^*−/−*^ mice, this suggests that CQ is capable of rescuing social interaction deficits in two independent mouse models of ASD characterized by reduced NMDAR function.

### CQ restores NMDAR function at *Tbr1* ^*+/−*^ amygdalar synapses

We hypothesized that the CQ-dependent social rescue in *Tbr1*^*+/−*^ mice might be associated with the restoration of reduced NMDAR function at *Tbr1*^*+/−*^ synapses. We first examined synaptic transmission at *Tbr1*^*+/−*^ synapses in the hippocampus, where reduced NMDAR function was observed in *Shank2*^*−/−*^ mice. However, no significant differences could be observed in the electrophysiological parameters, including miniature EPSCs (mEPSCs) in CA1 pyramidal neurons, and input–output ratio, paired-pulse ratio and NMDA/AMPA ratio at SC-CA1 synapses (Supplementary Fig. 12).

**Figure 8:**
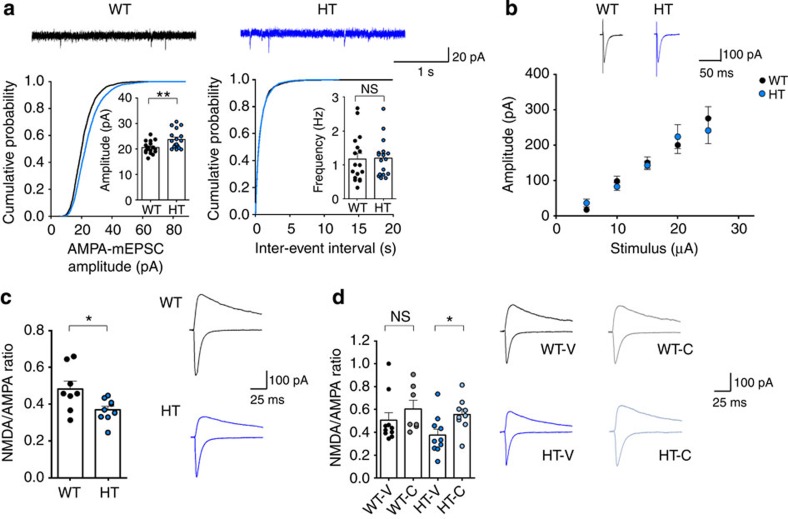
CQ restores NMDAR function at ***Tbr1***^***+/−***^ **amygdalar thalamic-LA (T-LA) synapses.** (**a**) CQ increases mEPSC amplitude but not frequency in *Tbr1*^*+/−*^ principal neurons in the LA (4–6 weeks). (*n*=17 cells (3 animals) for WT and HT, **P<0.01, Student's *t*-test). (**b**) CQ has no effect on the input–output ratio at *Tbr1*^*+/−*^ T-LA synapses (4–6 weeks), as indicated by plots of fEPSP slopes against stimulus intensities. Representative current traces are an average of three consecutive responses with input stimulations of 25 μA. (*n*=8 cells (3 animals) for WT and HT; Student's *t*-test). (**c**) Reduced NMDA/AMPA ratio at *Tbr1*^*+/−*^ T-LA synapses (4–6 weeks). (*n*=8 cells (5 animals) for WT and 9 (5) for HT; *P<0.05; Student's *t*-test). (**d**) CQ restores the NMDA/AMPA ratio at *Tbr1*^*+/−*^ T-LA synapses but has no effect on WT synapses (4–6 weeks). (*n*=10 (5) for WT-V, 7 (4) for WT-C, 10 (6) for HT-V and 9 (4) for HT-C; *P<0.05; two-way analysis of variance (ANOVA) and one-way ANOVA with Tukey's *post hoc* test). Data in all panels with error bars represent mean±s.e.m. NS, not significant.

In contrast, principal neurons in the lateral amygdala (LA), a brain region also enriched with glutamate- and Zn-releasing neurons^62^, showed slightly increased mEPSC amplitude but not frequency, without a change in the input–output ratio (Fig. 8a,b). Importantly, thalamic-LA *Tbr1*^*+/−*^ synapses showed a reduction in the NMDA/AMPA ratio, which was normalized by CQ treatment (Fig. 8c,d). CQ did not cause a significant increase in the NMDA/AMPA ratio in WT mice. These results suggest that *Tbr1* heterozygosity causes NMDAR hypofunction selectively in the amygdala, and its normalization is associated with the CQ-dependent social rescue in *Tbr1*^*+/−*^ mice.

## Discussion

In the present study, we found that trans-synaptic Zn mobilization improves social interaction in two distinct mouse models of ASD through postsynaptic Src and NMDAR activation.

Our study suggests that CQ-dependent mobilization of Zn from pre- to postsynaptic sites—not Zn removal after chelation—might be useful in the treatment of ASDs. This unique trans-synaptic Zn mobilization is supported by the following findings: (1) CQ failed to enhance NMDAR function in *ZnT3*^*−/−*^ mice, which lack the presynaptic Zn pool; and (2) Ca-EDTA, a membrane-impermeable Zn chelator that should chelate Zn in the synaptic cleft or extracellular sites, blocked CQ-dependent NMDAR activation.

CQ can bind Cu and Fe in addition to Zn. However, CQ appears to exert its effects through Zn interaction. A previous study has shown that CQ can mobilize Zn and Cu but not Fe into cytoplasmic sites in neuroblastoma cells^63^. In addition, our study shows that cuprizone, a specific Cu chelator, does not block CQ-dependent NMDAR activation. Regarding the potential involvement of the ‘chelating' activity of CQ, as opposed to the ‘ionophoric' activity, we suspect it is unlikely because CQ treatment of WT mice for 2 h did not lead to the reduction of Zn, Cu or Fe in the brain. However, it should be pointed out that the proposed Zn mobilization by CQ should involve Zn chelation at presynaptic sites before Zn ions are mobilized to postsynaptic sites through ionophoric effects.

We propose a specific mechanism that may underlie the CQ-dependent social rescue, namely NMDAR activation through postsynaptic Src. In support of this, CQ-dependent NMDAR activation at *Shank2*^*−/−*^ hippocampal synapses is blocked by two independent inhibitors of SFKs (PP2 and SU6656), as well as the Src-inhibitory peptide Src(40–58), which acts in the postsynaptic compartments when applied through patch pipettes. In addition, CQ treatment increases Src phosphorylation at Y416 but not Y527. We initially expected that the phosphorylation at Src Y527, which keeps Src at an inactive conformation, may be reduced to activate Src because Y527 is the substrate of Zn-inhibited C-terminal Src kinase^59^. On the contrary, we found an increase in the phosphorylation of Src Y416, which is known to render Src fully active. Although further details remain to be studied, CQ appears to promote full activation of Src after its initial activation by some other mechanisms.

Our study does not exclude the possibility that postsynaptic Zn acts on targets other than SFKs and NMDARs. For instance, a previous study has shown that Zn enhances excitatory synaptic stabilization of Shank2/3 and synapse formation and maturation^30^. Because two independent inhibitors of SFKs and the Src-inhibitory peptide significantly blocked the CQ-dependent NMDAR activation (Fig. 5), we did not explore this possibility. It should be noted, however, that the increase in CQ-induced NMDA-fEPSPs caused by additional Zn (250 nM) was greater at WT synapses than at *Shank2*^*−/−*^ synapses (Fig. 3d,e). This suggests that postsynaptic Shank2 may contribute to Zn-dependent NMDAR activation during high levels of neuronal activity and thus may also contribute to the aetiology of ASDs.

Previous studies have reported the roles of CQ in the regulation of synaptic transmission, including those from Dr Takeda's group^57^,^58^. The latter studies suggest that an increase in intracellular Zn concentration in the postsynaptic side of CA1 synapses inhibits NMDAR-dependent LTP, which apparently differ from our results that CQ has no effect on LTP at WT SC-CA1 synapses, and that CQ enhances LTP at *Shank2*^*−/−*^ SC-CA1 synapses (Fig. 2b). However, because *Shank2*^*−/−*^ synapses display substantial alterations in synaptic protein composition/modification and synaptic transmission/plasticity^45^, the CQ-dependent NMDAR activation at *Shank2*^*−/−*^ synapses cannot be compared with the previous results. Regarding the effect of CQ on LTP at WT synapses, the difference might stem from that (1) previous studies used rat hippocampal slices, whereas we used mouse ones, and (2) previous studies prepared brain slices 2 h after i.p. injection of Zn-CQ (9.8 μmol (∼3 mg) kg^−1^) and measured LTP without CQ in the artificial CSF (ACSF), whereas we introduced CQ (4 μM) directly to slices obtained from CQ-untreated mice during electrophysiological measurements. Notably, an independent paper has also reported that bath application of CQ (4 μM) has no effect on LTP at SC-CA1 synapses in the mouse brain, similar to our data^63^. To address this issue directly, we mimicked the method of CQ treatment reported in Dr Takeda's group, preparing mouse hippocampal slices 2 h after i.p. injection of Zn-CQ (9.8 μmol kg^−1^). However, we found no difference between treated and untreated groups in LTP induced by high-frequency stimulation at SC-CA1 synapses (*n*=7 slices, 4 mice for vehicle and 7, 5 for CQ; data not shown). Therefore, the differences may be attributable to the fact that different animal species were used.

CQ-dependent rescue of social deficits in *Shank2*^*−/−*^ and *Tbr1*^*+/−*^ mice was associated with CQ-dependent elevation of NMDAR function, further supporting the hypothesis that NMDAR hypofunction may underlie ASDs. This concept was put forward based on the observations that D-cycloserine improves ASD symptoms in humans and autistic-like phenotypes in animals (reviewed in ref. 64), although further studies are needed to verify this hypothesis.

CQ rescues social interaction in *Shank2*^*−/−*^ and *Tbr1*^*+/−*^ mice, but it fails to rescue social novelty recognition, repetitive behaviour, hyperactivity or anxiety-like behaviour in *Shank2*^*−/−*^ mice. This is reminiscent of the previous result that NMDAR activation by D-cycloserine selectively rescues social interaction in *Shank2*^*−/−*^ mice^45^. This selective rescue might be attributable to the different nature of the circuits associated with these behaviours, where some are reversible, or at least treatable, whereas others are not. In line with this, NMDARs are involved in the regulation of both neuronal development and synaptic transmission/plasticity/signalling^11^,^65^. In addition, activity-dependent sculpting of neuronal circuits associated with ASDs has critical time windows^66^.

Finally, our study broadens the therapeutic potential of CQ. CQ has been used as a topical antiseptic or an oral intestinal amoebicide since 1930s, although the latter use has ceased for its controversial association with subacute myelo-optic neuropathy^52^. Recently, however, CQ-dependent chelation of Zn has been suggested for the treatment of neurological disorders including Alzheimer's disease^67^, Parkinson's disease^68^ and Huntingtons' disease^69^. Moreover, PBT2, a second-generation CQ-related compound under clinical trials, seems to be safe and improve cognitive deficits in patients with Alzheimer's disease^67^. Therefore, our study is the first to demonstrate the possibility of repositioning of the FDA-approved antibiotic, CQ, to ASDs based on a novel mechanism distinct from chelation. In addition, CQ-dependent trans-synaptic Zn mobilization might also be useful in other psychiatric disorders that are notable for being caused by a decrease in NMDAR function^70^.

In conclusion, our study suggests that trans-synaptic Zn mobilization rapidly improves social interaction in two independent mouse models of ASD through Src and NMDAR activation, and a new therapeutic potential of CQ in the treatment of ASDs.

## Methods

### Mice

*Shank2*^*−/−*^ mice and *Tbr1*^*+/−*^ mice have been reported^45^,^50^. All mice were backcrossed to a C57BL/6 background for more than 20 generations, and housed and bred in a mouse vivarium at the Korea Advanced Institute of Science and Technology (KAIST; *Shank2*^*−/−*^ mice) and the Academia Sinica (*Tbr1*^*+/−*^ mice). For breeding of *Shank2* mice, we used a scheme of heterozygous (HT) × HT to produce littermate pairs of WT and KO mice. To breed *Tbr1* mice, we used offspring from HT males × WT females. Other combinations did not yield any differences in breeding efficiency or behavioural phenotypes. Pups were kept with the dam until weaning at postnatal day 21. After weaning, animals were housed in mixed-genotype groups of 3–5 mice per cages, and randomly subjected to electrophysiological and behavioural experiments. Animals at 3–5 weeks of age were used for electrophysiological experiments and two-photon imaging; male animals at 2–4 months of age were used for behavioural assays. For TFL-Zn staining, male animals at 8 weeks (for *Shank2*^*−/−*^ mice) were used. WT littermates were used as controls.

*ZnT3*^*−/−*^ mice, reported previously^54^, were maintained in the KAIST animal facility. These mice also were backcrossed to the C57BL/6 background for more than 10 generations. Both male and female animals at 3–5 weeks were used for electrophysiological experiments and TFL-Zn staining (P23).

All mice were bred and maintained according to the KAIST and Academia Sinica Animal Research Requirements, and all procedures were approved by the Committees of Animal Research at KAIST, and at Academia Sinica. Mice were fed *ad libitum* by standard rodent chow and tap water, and housed under 12 h light/dark cycle (lights off at 1900 hours in KAIST and at 2000 hours in Academia Sinica).

### Clioquinol

CQ (Calbiochem) was dissolved in dimethylsulfoxide (DMSO; Sigma) and polyethylene glycol (Aldrich; DMSO:polyethylene glycol=1: 9) to a final concentration of 20 g l^−1^. WT and *Shank2*^*−/−*^ mice (or *Tbr1*^*+/−*^ mice) received acute i.p. injection of CQ (30 mg kg^−1^) or the same volume of DMSO-polyethylene glycol mixture. The injection was performed 2 h before (*Shank2*^*−/−*^ mice and WT littermates) or 3 h before (*Tbr1*^*+/−*^ mice and WT littermates) behavioural assays at the discretion of the facility.

### Drug treatment scheme

We devised a within-subjects design with a 1-week washout period (Supplementary Fig. 1a), and divided animals into two groups, vehicle-first and CQ-first group, to rule out carryover effects. Each mouse received a single acute dose of vehicle or CQ, and underwent a single behavioural task, one task per week. Testing was conducted in dedicated behavioural test rooms during the light phase (three-chamber test) and the dark phase (repetitive behaviours and open-field test).

### Three-chamber social interaction assay

The three-chamber social interaction assay for *Shank2* mice (*Shank2*^*−/−*^ and WT littermates) and *Tbr1* mice (*Tbr1*^*+/−*^ and WT littermates) were performed^45^,^50^. In short, the assay consisted of three phases of 10 min duration: habituation, social interaction (stranger 1 versus object) and social novelty recognition (stranger 1 versus stranger 2). Exploration was defined as instances in which WT or mutant mouse tries to sniff object/stranger, or orients its nose towards and come close to object/stranger. Individual movement tracks were analysed by Ethovision 10.0 (Noldus) and modified by custom-designed software MatLab (MathWorks) to generate heat maps. Time spent in exploration was analysed by the researcher who was blinded to the subject genotype (in *Shank2* mice), or by using the Smart Video Tracking System (Panlab, in *Tbr1* mice). In addition to exploration time, we used the preference index, which represents a numerical difference between time spent exploring the targets (stranger 1 versus object or stranger 2 versus stranger 1) divided by total time spent exploring both targets^45^.

### Repetitive behaviours

*Shank2*^*−/−*^ mice and their WT littermates in their home cages without bedding were used to measure times spent in repetitive behaviours, including jumping and grooming during 10 min. Jumping was defined as the behaviour of a mouse where it rears on its hind legs at the corner of the cage, or along the side walls, and jumps so that the two hind legs are simultaneously off the ground. Grooming behaviour was defined as stroking or scratching of face, head or body with the two forelimbs, or licking body parts^45^. The experiments and analyses were performed independently in a blind manner.

### Open-field test

The size of the open-field box was 40 × 40 × 40 cm, and the centre zone line was 13.3 cm apart from the edge. Mice were placed in the centre in the beginning of the test, and mouse movements were recorded with a video camera for 60 min, and were analysed by Ethovision 10.0 (Noldus).

### Electrophysiology

For hippocampal electrophysiological experiments, sagittal hippocampal slices (400 μm thick for extracellular and 300 μm thick for intracellular recordings) of the mutant mice (*Shank2*^*−/−*^ mice, *Tbr1*^*+/−*^ mice or *ZnT3*^*−/−*^ mice) and their WT littermates were prepared using a vibratome (Leica VT1200) in ice-cold dissection buffer containing (in mM) 212 sucrose, 25 NaHCO_3_, 5 KCl, 1.25 NaH_2_PO_4_, 0.5 CaCl_2_, 3.5 MgSO_4_, 10 D-glucose, 1.25 L-ascorbic acid and 2 Na-pyruvate bubbled with 95% O_2_/5% CO_2_. CA3 was removed to prevent epileptiform activity. For amygdalar electrophysiological experiments, coronal slices (300 μm) including the LA of *Tbr1*^*+/−*^ mice and their WT littermates were cut. The slices were recovered at 32 °C for 1 h in normal ACSF (in mM: 124 NaCl, 2.5 KCl, 1 NaH_2_PO_4_, 25 NaHCO_3_, 10 glucose, 2 CaCl_2_ and 2 MgSO_4_ oxygenated with 95% O_2_/5% CO_2_). For the recording, a single slice was moved to and maintained in submerged-type chamber at 28 °C, continuously perfused with ACSF (2 ml min^−1^) saturated with 95% O_2_/5% CO_2_. Stimulation and recording pipettes were pulled from borosilicate glass capillaries (Harvard Apparatus) using a micropipette electrode puller (Narishege).

For extracellular recordings, mouse hippocampal slices at the age of postnatal day 21–35 were used. fEPSPs were recorded in the stratum radiatum of the hippocampal CA1 region using pipettes filled with ACSF (1 MΩ). fEPSP was amplified (Multiclamp 700B, Molecular Devices) and digitized (Digidata 1440A, Molecular Devices) for measurements. The Schaffer collateral pathway was stimulated every 20 s with pipettes filled with ACSF (0.3–0.5 MΩ). The stimulation intensity was adjusted to yield a half-maximal response, and three successive responses were averaged and expressed relative to the normalized baseline. To induce LTP, high-frequency stimulation (100 Hz, 1 s) was applied after a stable baseline was acquired. CQ (4 μM) was bath applied before and after LTP induction during the whole experimental processes. To isolate NMDAR-mediated fEPSPs, we used ACSF containing 2 mM calcium, 0.1 mM magnesium and 6,7-dinitroquinoxaline-2,3-dione (10 μM, DNQX, Tocris), which inhibits AMPAR-mediated EPSPs.

Whole-cell patch-clamp recordings of hippocampal CA1 pyramidal neurons, and of LA principal neurons in the dorsolateral division were made using a MultiClamp 700B amplifier (Molecular Devices) and Digidata 1440A (Molecular Devices). During whole-cell patch-clamp recordings, series resistance was monitored each sweep by measuring the peak amplitude of the capacitance currents in response to short hyperpolarizing step pulse (5 mV, 40 ms); only cells with a change in <20% were included in the analysis. For afferent stimulation of hippocampal pyramidal neurons, the Schaffer collateral pathway was selected, while for that of LA, the thalamic afferent pathway was stimulated. For LA electrophysiology, brain slices were selected based on the presence of a well-isolated, sharply defined trunk (containing thalamic afferents) crossing the dorsolateral division of the LA, which is a site of convergence of somatosensory and auditory inputs. For NMDA/AMPA ratio experiments, mouse hippocampal slices (P17–P25) and LA slices (4–6 weeks old) were used. The recording pipettes (2.5–3.5 MΩ) were filled with an internal solution containing the following (in mM): 100 CsMeSO_4_, 10 TEA-Cl, 8 NaCl, 10 HEPES, 5 QX-314-Cl, 2 Mg-ATP, 0.3 Na-GTP and 10 EGTA, with pH 7.25, 295 mOsm). CA1 pyramidal neurons and LA principal neurons were voltage clamped at −70 mV, and EPSCs were evoked at every 15 s. AMPAR-mediated EPSCs were recorded at −70 mV, and 30 consecutive responses were recorded after stable baseline. After recording AMPAR-mediated EPSCs, holding potential was changed to +40 mV to record NMDAR-mediated EPSCs. NMDA component was measured at 60 ms after the stimulation. The NMDA/AMPA ratio was determined by dividing the mean value of 30 NMDA components of EPSCs by the mean value of 30 AMPAR-mediated EPSC peak amplitudes. Somatic whole-cell recording of mEPSCs were obtained in amygdalar principal neurons at a holding potential of −70 mV. TTX (1 μM) and picrotoxin (100 μM) were added to ACSF to inhibit spontaneous action potential-mediated synaptic currents and IPSCs, respectively. CQ (4 μM) was bath applied from 20 min before and during the whole period of NMDA/AMPA ratio recording. For measuring AMPAR-mediated and NMDAR-mediated EPSCs together upon CQ treatment, pyramidal neurons were voltage clamped at −40 mV, and EPSCs were evoked at every 15 s. AMPAR-mediated EPSC was determined as a peak amplitude of EPSC, and NMDAR-mediated EPSC as a component at 60 ms after stimulation. NMDA/AMPA ratio at −40 mV was calculated by using both values, and monitored during the experimental process. Src-inhibiting peptide, Src(40–58), and its analogue, scrambled Src(40–58; ref. 61), were purchased from Peptron, and introduced into the internal solution at a concentration of 0.03 mg ml^−1^ to observe whether Src-inhibition affects the action of CQ. Picrotoxin (100 μM) was always added to ACSF to block GABAA receptor-mediated currents.

Data were acquired by Clampex 10.2 (Molecular Devices) and analysed by Clampfit 10 (Molecular Devices). Drugs were purchased from Tocris (DNQX, TPEN, PP2, PP3 and D-AP5), Abcam (PD98059) and Sigma (KN93, picrotoxin, Ca-EDTA, cuprizone and SU6656).

### ZnAF-2DA imaging

For intracellular Zn staining, two groups of WT mice (3–5 weeks) were injected with vehicle (DMSO) or CQ (30 mg kg^−1^) 2 h before imaging. For two-photon imaging, sagittal hippocampal slices (300 μm thick) were immersed in 10 μM ZnAF-2DA (Enzo Life Sciences) in ACSF for 40 min, followed by 1 h washout with ACSF. Zn signals were measured by using a multiphoton laser scanning microscope system LSM 7 MP (Carl Zeiss). A total of ∼200 Z stack images (0.7 μm interval; ∼130–180 μm total depth) were captured from each slice. For quantification of Zn signals, a total of 11 images at every 4.5 μm depth from the surface (from∼5 to 50 μm depth) were selected. Three regions of interest (ROIs; squares of 33 μm^2^) in the CA1 cell body area, or in the CA1 dendritic field, were analysed of Zn fluorescence signals using MetaMorph (Molecular Devices).

### TFL-Zn staining

Without fixation, brain sections (10 μm thick) were stained with the Zn-specific dye TFL-Zn (*N*-(6-methoxy-8-quinolyl)-p-carboxybenzoylsulfonamide (0.1 mM), Calbiochem) dissolved in phosphate-buffered saline (pH 7.2), and photographed with a digital camera linked to a fluorescence microscope (Olympus IX71; excitation, 330–385 nm; dichromatic, 400 nm; and barrier, 420 nm). Fluorescence signals were obtained using an image programme (Image-Pro Insight, Media Cybernetics, Silver Spring, MD). Images of five consecutive hippocampal slices from an individual brain were quantified using MetaMorph (Molecular Devices). ROIs were defined as three to five squares (50 × 50 μm^2^) in WT and *Shank2*^*−/−*^ hippocampal DG, CA3 and CA1 regions (5, 3 and 5 squares, respectively). For quantification, the total fluorescence from a *Shank2*^*−/−*^ ROI was normalized to that from an equivalent WT ROI.

### Src immunoblot analysis

For immunoblotting of Src proteins, WT and *Shank2*^*−/−*^ sagittal hippocampal slices (400 μm thick; 3–5 weeks) were prepared using a vibratome (Leica VT1200). After recovery at 37 °C for 1 h in normal ACSF supplemented with 250 nM ZnCl_2_, slices were treated with CQ, or vehicle (DMSO), for 20 min, in the presence or absence of PP2. After treatments, the slices were homogenized in 100 μl ice-cold homogenization buffer (0.32 M sucrose, 10 mM HEPES, pH 7.4, 2 mM EDTA, protease inhibitors and phosphatase inhibitors) per each slice, and subjected to immunoblot analysis and quantification using Odyssey Fc Imaging System (LI-COR). The following antibodies were purchased: Src, phosphor-Src (Tyr-416; 1:1,000 dilution) and phosphor-Src (Tyr-527; Cell Signaling; 1:1,000 dilution). Full-size immunoblot images for Fig. 6 are shown in Supplementary Fig. 13.

### Crude synaptosomes

Crude synaptosomes from *Shank2*^*−/−*^ mice were prepared as described^45^. Briefly, mouse brains (2 months old) were homogenized in ice-cold homogenization buffer (0.32 M sucrose, 10 mM HEPES, pH 7.4, 2 mM EDTA, protease inhibitors and phosphatase inhibitors). The homogenates were centrifuged at 900*g* for 10 min. The resulting supernatant was centrifuged again at 12,000*g* for 15 min. The pellet was resuspended in homogenization buffer and centrifuged at 13,000*g* for 15 min (the resulting pellet is P2; crude synaptosomes). This sample was immunoblotted with ZnT3 antibodies (SYSY).

### Metal analysis

WT and *Shank2*^*−/−*^ mice (2*–*3 months) were treated with CQ (30 mg kg^−1^), or DMSO, by i.p. injection 2 h before brain preparation and subsequent whole-brain metal analysis by inductively coupled plasma mass spectrometry.

### Statistical analysis

We randomly performed all the behaviour experiments, ZnAF-2DA and TFL-Zn imaging, and metal analysis by researchers blind to the identity of the animals, and analysed the data in a blind manner. Data collection and analysis for slice electrophysiology and immunoblotting were performed randomly, but not blind to the conditions of the experiments. Statistical analyses were performed using Prism GraphPad (version 6.05) software, and details of the results are described in Supplementary Data 1. No statistical methods were used to predetermine sample sizes, but our sample sizes are similar to those reported in previous related publications^45^,^50^. An outlier was defined as a value outside the mean±3 s.d.

## Additional information

**How to cite this article:** Lee, E. -J. *et al*. Trans-synaptic zinc mobilization improves social interaction in two mouse models of autism through NMDAR activation. *Nat. Commun.* 6:7168 doi: 10.1038/ncomms8168 (2015).

## Supplementary Material

Supplementary Information Supplementary Figures 1-13

Supplementary Movie 1 Zn signals in vehicle-treated hippocampal slices.

Supplementary Movie 2 Zn signals in CQ-treated hippocampal slices.

Supplementary Data 1 Detailed statistical analysis.
