# Supplementary material for: Trans-synaptic zinc mobilization improves social interaction in two mouse models of autism through NMDAR activation
Source: Nat Commun. 2015 May 18;6:7168. doi: 10.1038/ncomms8168 (PMC4479043; doi:10.1038/ncomms8168)
Supplement: Supplementary Information — Supplementary Figures 1-13 [file ncomms8168-s1.pdf]

Trans-synaptic Zinc mobilization improves social interaction in two mouse models of autism through NMDAR activation

Eun-Jae Lee,[1,3] Hyejin Lee,[2,3] Tzyy-Nan Eugene Huang,[4] Changuk Chung,[2,3] Wangyong Shin, [2,3] Kyungdeok Kim, [2,3] Jae-Young Koh,[5,6,7] Yi-Ping Hsueh,[4,*] and Eunjoon Kim [2,3,*]

[1]Graduate School of Medical Science and Engineering, Korea Advanced Institute of Science and Technology (KAIST), Daejeon 305-701, Korea; [2]Department of Biological Sciences, Korea Advanced Institute of Science and Technology (KAIST), Daejeon 305-701, Korea; [3]Center for Synaptic Brain Dysfunctions, Institute for Basic Science (IBS), Daejeon 305-701, Korea; [4]Institute of Molecular Biology, Academia Sinica, Taipei, 115, Taiwan; [5]Neural Injury Research Lab, University of Ulsan College of Medicine, Seoul, Korea; [6]Asan Institute for Life Science, University of Ulsan College of Medicine, Seoul, Korea; [7]Department of Neurology, University of Ulsan College of Medicine, Seoul, Korea; *Corresponding authors.

Correspondence: kime@kaist.ac.kr (E.K.), yph@gate.sinica.edu.tw (Y.-P.H)

Supplementary figures

Suppl Fig 1

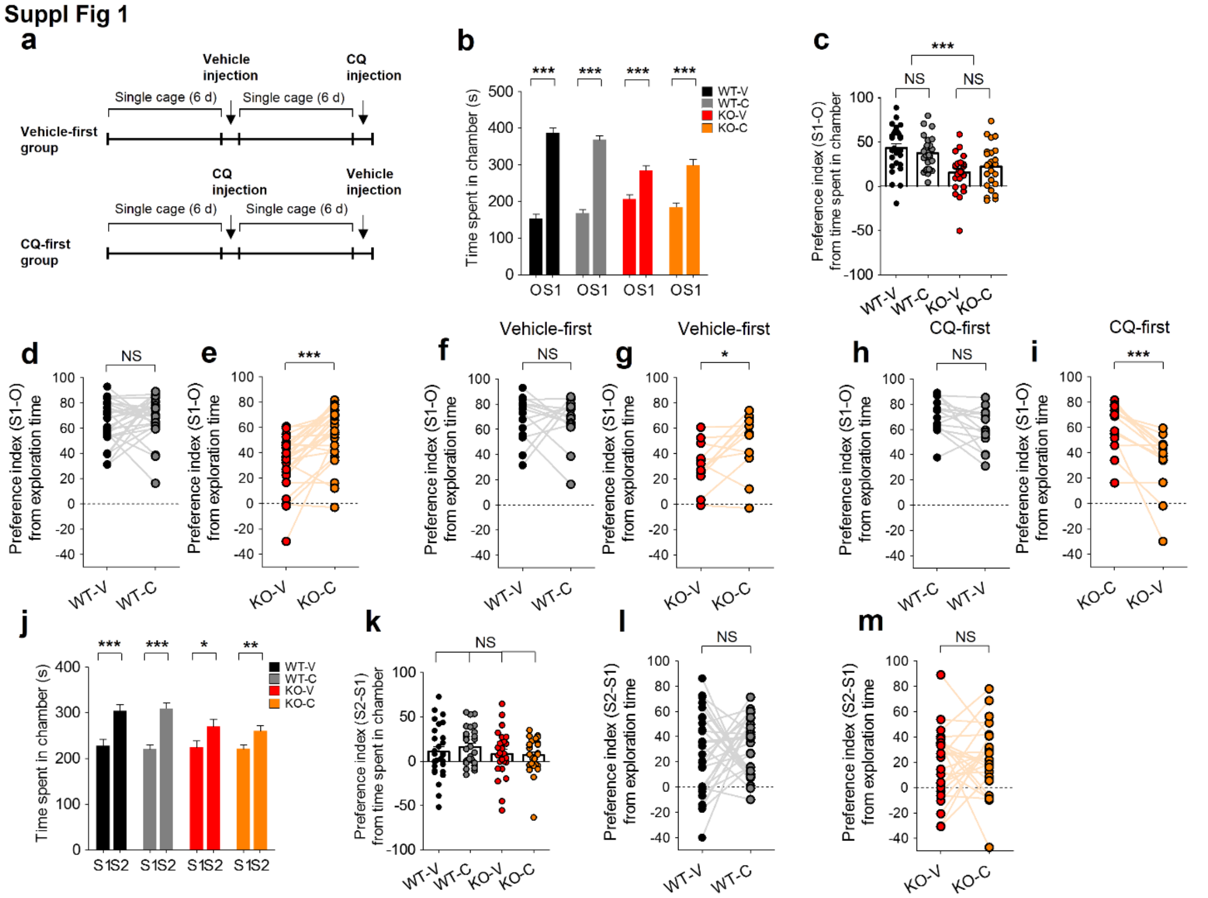

Supplementary Fig. 1. CQ improves social interaction in *Shank2*$^{-/-}$ mice

(**a**) A schematic depicting drug-administration paradigms. Animals were divided into two experimental groups; one received vehicle first and CQ second, and the other received CQ first and vehicle second. Mice were tested 2 hours after receiving the first injection (vehicle or 30 mg kg^{-1} CQ; i.p.). After a 6-day rest period in single cages, mice received the reciprocal treatment and were retested.

(**b-m**) CQ improves social interaction (b-i) but has no effect on social novelty recognition (j-m) in *Shank2*$^{-/-}$ (KO) mice, or on both social interaction and social novelty recognition in WT mice, as determined by time spent in exploration, preference index from exploration time, and time spent exploring/sniffing targets (S1/stranger vs. O/object, or S2/new stranger vs. S1/previous stranger). Data were analyzed as paired comparisons of the effects of CQ (before and after) within WT and KO groups, or within the vehicle-first and CQ-first groups to minimize carryover effects. (n = 28 for WT-V and WT-C, 25 for KO-V and KO-C, n = 14 for WT-V and WT-C (vehicle-first), n = 12 for KO-V and KO-C (vehicle-first), n = 14 for WT-V and WT-C (CQ-first), n = 13 for KO-V and KO-C (CQ-first), NS, not significant, *p < 0.05, **p < 0.01, ***p < 0.001, Student's t-test). Data in all panels with error bars represent mean ± s.e.m.

Suppl Fig 2

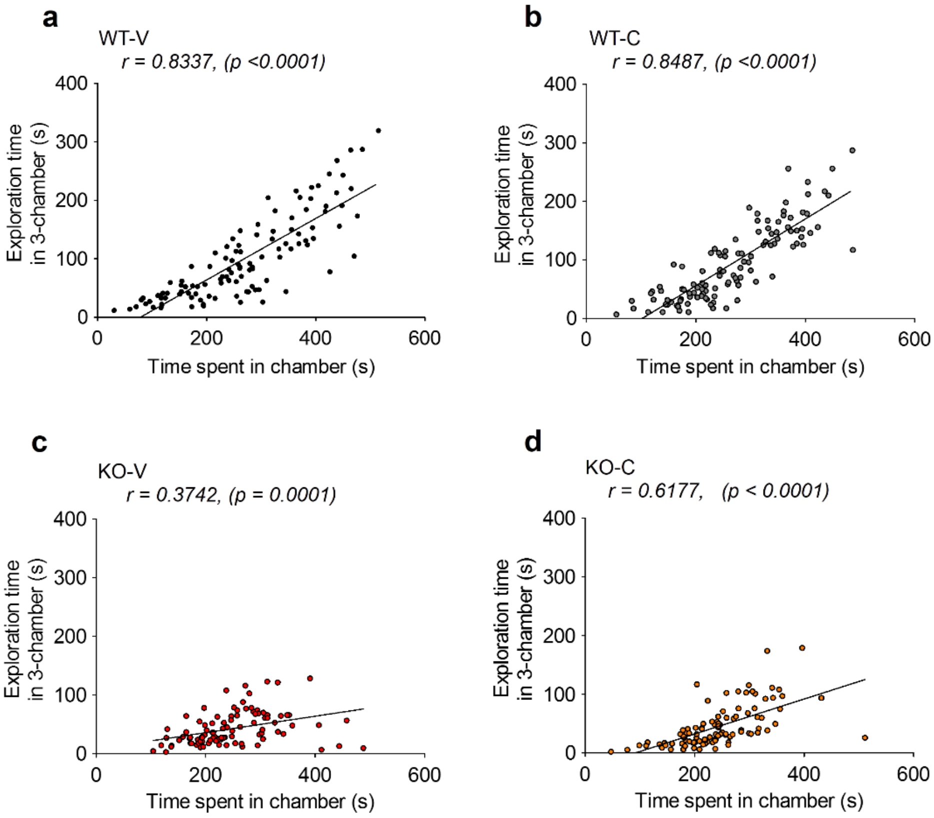

Supplementary Fig. 2. Limited correlation between chamber time and exploration time in *Shank2*$^{-/-}$ mice, relative to WT mice.

(**a-d**) Time spent in chamber (chamber time) and time spent in exploring/sniffing the targets (O, S1, and S2) (exploration time) do not correlate well in *Shank2*$^{-/-}$ mice, relative to WT mice, as determined using the data from the three-chamber social interaction results from **Fig. 1** and **Supplementary Fig. 1**. Note that this limited correlation in *Shank2*$^{-/-}$ mice is mildly improved by CQ treatment (Pearson correlation).

Suppl Fig 3

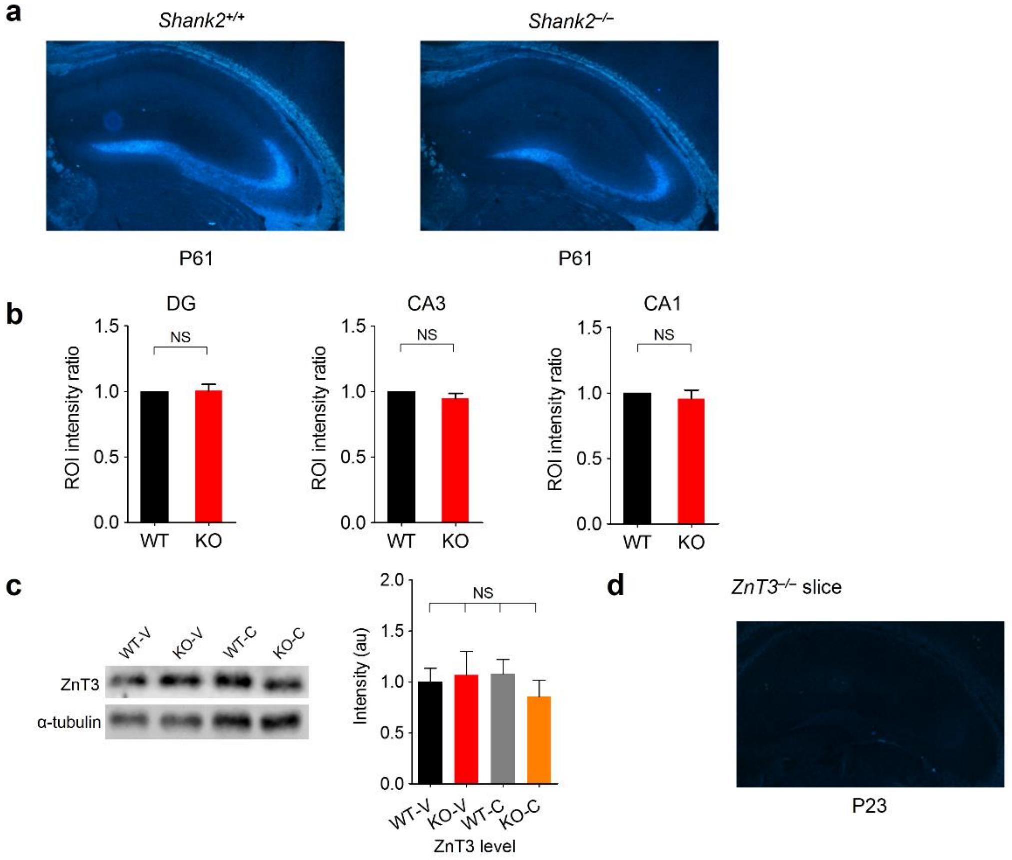

Supplementary Fig. 3. Free Zn levels are similar in WT and *Shank2*$^{-/-}$ brains, and CQ has no effect on ZnT3 protein levels

(**a** and **b**) There was no difference in the total level of free Zn between WT and *Shank2*$^{-/-}$ brains (8 wks), as determined using the free Zn-binding fluorescent dye, TFL-Zn. (n = 15 hippocampal slices from 3 animals for WT and KO, NS, not significant, Student's t-test). ROI, region of interest.

(**c**) CQ treatment has no effect on the total level of ZnT3 protein in whole brain crude synaptosomal fractions from WT and *Shank2*$^{-/-}$ mice (8 wks). Mice were acutely injected with CQ (30 mg kg^{-1}; i.p.) 2 hours before sample preparation and immunoblot analysis. (n = 4 for each group, NS, not significant, one-way ANOVA). Data in all panels with error bars represent mean ± s.e.m.

(**d**) Free Zn was undetectable in *ZnT3*$^{-/-}$ brain (postnatal day 23), as determined by TFL-Zn.

Suppl Fig 4

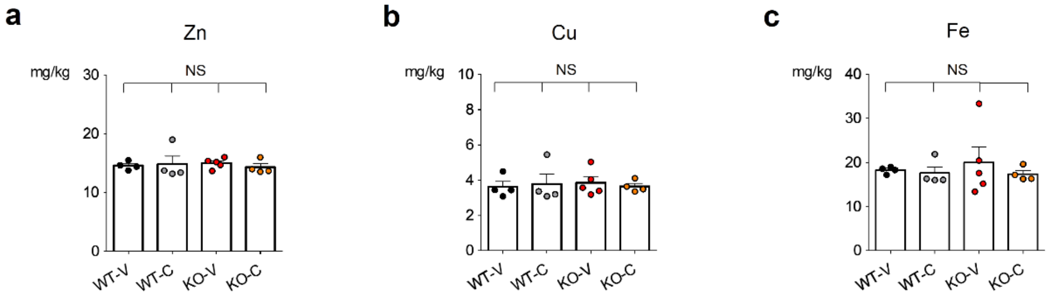

Supplementary Fig. 4. WT and *Shank2*$^{-/-}$ mice show comparable levels of Zn, Cu, and Fe in the brain, and CQ treatment has no effect on brain levels of these metals.

(**a**-**c**) WT and *Shank2*$^{-/-}$ mice (2–3 months) were treated with CQ (30 mg kg^{-1}), or vehicle (DMSO), by i.p. injection for 2 hrs, followed by whole-brain metal analysis by inductively coupled plasma mass spectrometry. (n = 4 for WT-V, 4 for WT-C, 5 for KO-V, and 4 for KO-C, NS, not significant, two-way ANOVA and one-way ANOVA with Tukey's *post hoc* test). Data in all panels with error bars represent mean ± s.e.m.

Suppl Fig 5

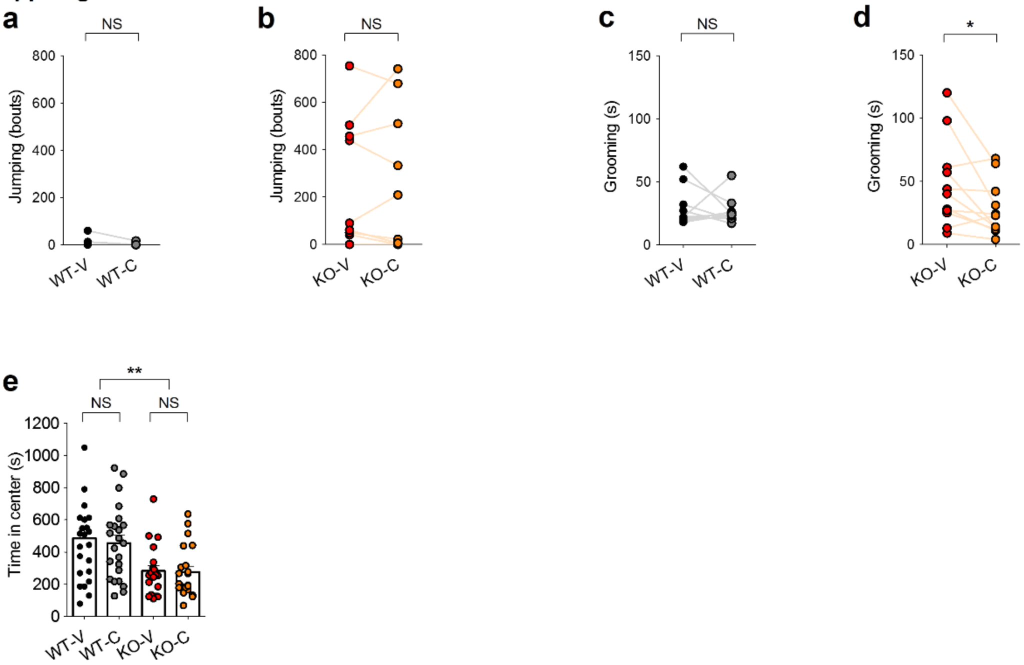

Supplementary Fig. 5. CQ fails to improve repetitive behavior and anxiety-like behavior in *Shank2*$^{-/-}$ mice

(**a-d**) CQ (30 mg kg^{-1}; i.p.) injected 2 hours prior to testing fails to improve jumping and has no effect on grooming in *Shank2*$^{-/-}$ mice. (n = 10 for WT-V and WT-C, 11 for KO-V and for KO-C, NS, not significant, *p < 0.05, Student's t-test)

(**e**) CQ has no effect on the time spent in the center region of the open field arena in WT and *Shank2*$^{-/-}$ mice. (n = 10 for WT-V and WT-C, 11 for KO-V and for KO-C, NS, not significant, *p < 0.05, two-way ANOVA and Kruskal-Wallis one-way ANOVA with Dunn's *post hoc* test) Data in all panels with error bars represent mean ± s.e.m.

Suppl Fig 6

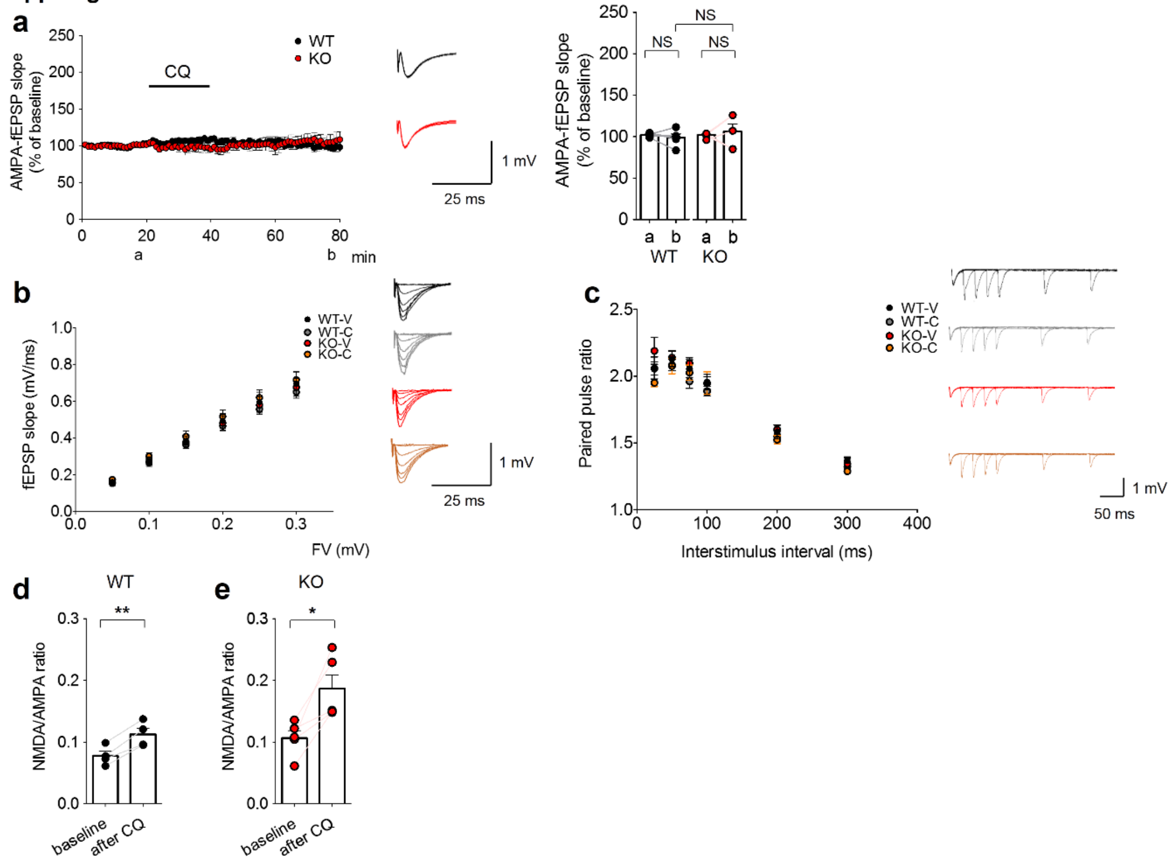

Supplementary Fig. 6. CQ has no effect on AMPA-fEPSPs, input-output ratio, or paired pulse ratio, but Increases the NMDA/AMPA ratio of eEPSCs at *Shank2*$^{-/-}$ synapses

(**a**) CQ (4 µM) has no effect on AMPA-fEPSPs. The labels a and b indicate 5-min duration before CQ and the end of recording, respectively. (n = 5 slices (4 animals) for WT and 5 (4) for KO, NS, not significant, Student’s t-test)

(**b**) CQ (4 µM) has no effect on the input-output relationship at WT or *Shank2*$^{-/-}$ hippocampal SC-CA1 synapses, as determined by plotting the initial slopes of AMPA-fEPSPs against amplitudes of fiber volley. (n = 9 slices (7 animals) for WT-V, 9 (7) for WT-C, 8 (5) for KO-V, and 9 (6) for KO-C, one-way ANOVA).

(**c**) CQ (4 µM) has no effect on the paired pulse ratio at both WT and *Shank2*$^{-/-}$ hippocampal SC-CA1 synapses, as determined by plotting the ratio of first/second initial slopes of AMPA-fEPSPs against interstimulus intervals. (n = 9 slices (7 animals) for WT-V, 9 (7) for WT-C, 8 (5) for KO-V, and 9 (6) for KO-C, one-way ANOVA).

(**d** and **e**) CQ (4 µM) increases the NMDA/AMPA ratio of eEPSCs at -40 mV in both WT and *Shank2*$^{-/-}$ hippocampal SC-CA1 synapses. (n = 4 cells (3 animals) for WT, and 5 (4) for KO, *p < 0.05, Student’s t-test). Data in all panels with error bars represent mean ± s.e.m.

Suppl Fig 7

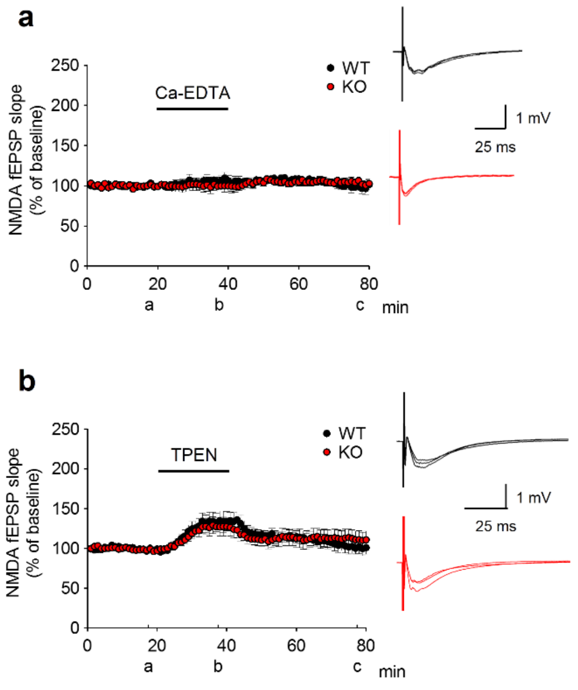

Supplementary Fig. 7. Ca-EDTA has no effect on the basal NMDAR function, while TPEN causes a small increase in NMDAR function.

(**a** and **b**) The effect of Ca-EDTA (2 mM) or TPEN (25 μM) on NMDA-fEPSPs. The labels a, b, and c indicate 5-min duration before and during Ca-EDTA, and at the end of recording, respectively. (n = 8 slices (5 animals) for WT-Ca-EDTA, 10 (5) for KO-Ca-EDTA, 11 (6) for WT-TPEN, and 9 (5) for KO-TPEN, NS, not significant, *p < 0.05, Repeated measures ANOVA). Data in all panels with error bars represent mean ± s.e.m.

Suppl Fig 8

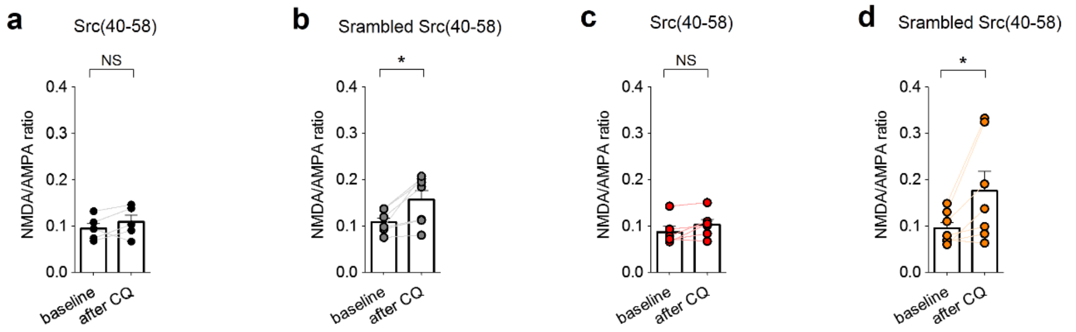

Supplementary Fig. 8. Src-inhibitory peptide blocks CQ-dependent NMDAR activation

(**a-d**) Src-inhibitory peptide, Src(40-58), but not its scrambled version, sSrc(40-58), blocks CQ-dependent NMDAR activation, as measured by the NMDA/AMPA ratio at -40 mV. (Src(40-58), n = 5 cells (4 animals) for WT and 6(4) for KO; sSrc(40-58), n = 7 (5) for WT and 7(6) for KO, NS, not significant, *p < 0.05, Student's t-test). Data in all panels with error bars represent mean ± s.e.m.

Suppl Fig 9

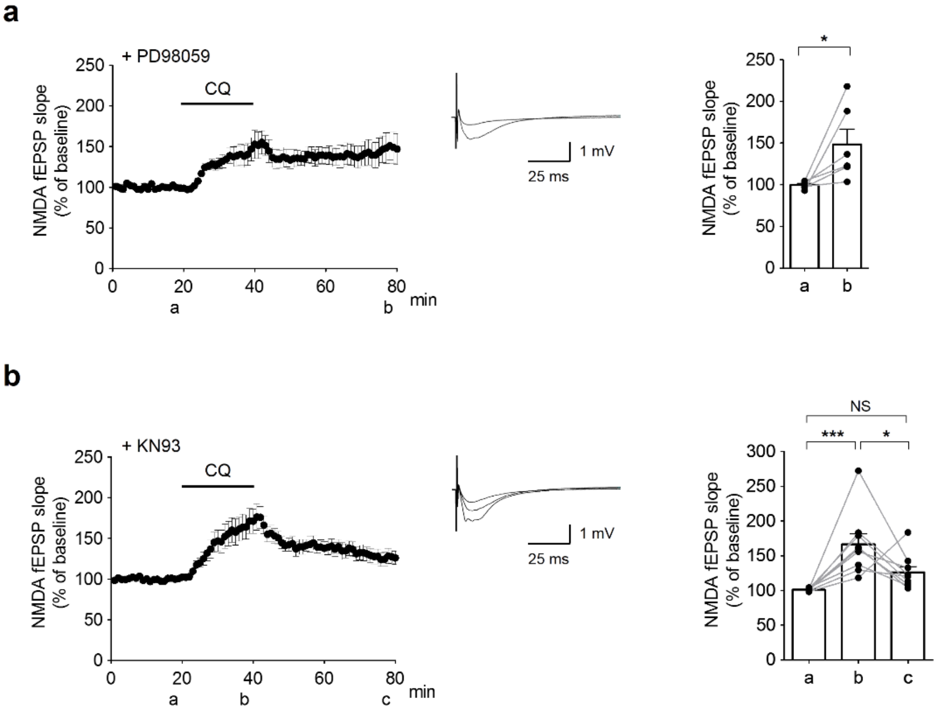

Supplementary Fig. 9. CaMKIIα is required for the maintenance of the enhanced NMDAR function induced by CQ.

(**a** and **b**) WT hippocampal slices were treated with CQ (4 μM) for 20 min in the presence of PD98059 (MAPKK/MEK inhibitor) or KN93 (CaMKIIα inhibitor) and measured of NMDA fEPSPs. (PD98059, n = 6 slices (4 animals); KN93, n = 9 (4), *p < 0.05, ***p < 0.001, Student's t-test and repeated measures ANOVA). Data in all panels with error bars represent mean ± s.e.m.

Suppl Fig 10

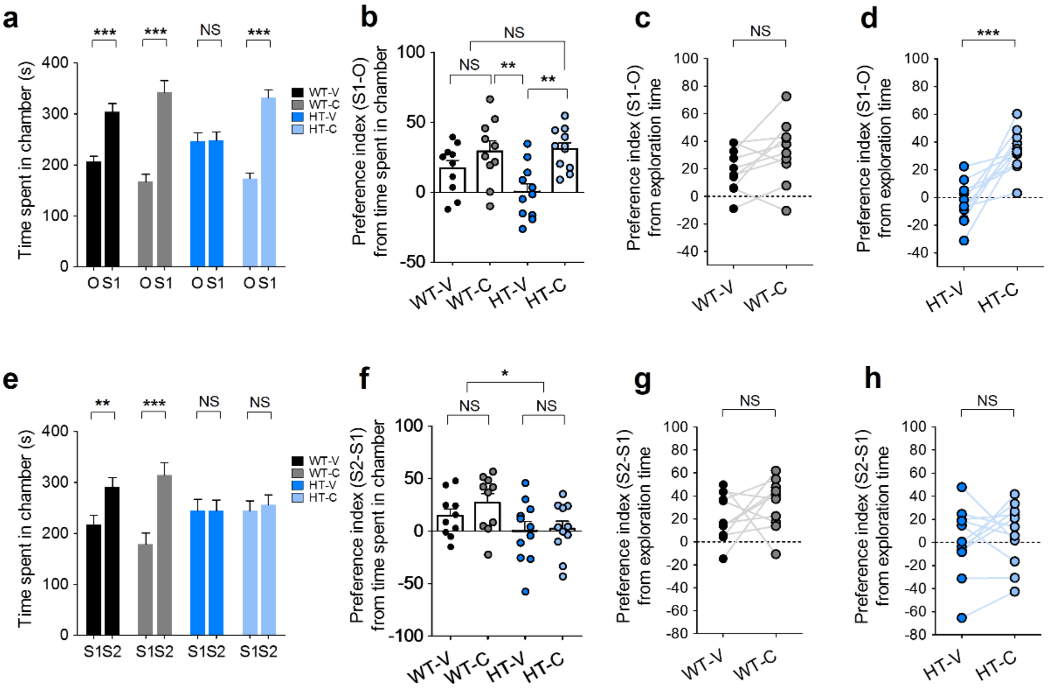

Supplementary Fig. 10. CQ improves social interaction in *Tbr1*$^{+/–}$ (HT) mice

(**a**-**h**) CQ improves social interaction (a-d) but has no effect on social novelty recognition (e-h) in *Tbr1*$^{+/–}$ mice, or on both parameters (social interaction and social novelty recognition) in WT littermates, as determined by the times spent in exploring/sniffing the targets (S1/strangers vs. O/object, or S2/new stranger vs. S1/old stranger). The paired comparisons of the effects of CQ (before and after) within the WT or HT group were made to minimize carryover effects. (n = 10 for WT-V and WT-C, n = 11 for HT-V and HT-C, NS, not significant, *p < 0.05, ***p < 0.001, Student's t-test, two-way ANOVA and one-way ANOVA with Tukey's *post hoc* test). Data in all panels with error bars represent mean ± s.e.m.

Suppl Fig 11

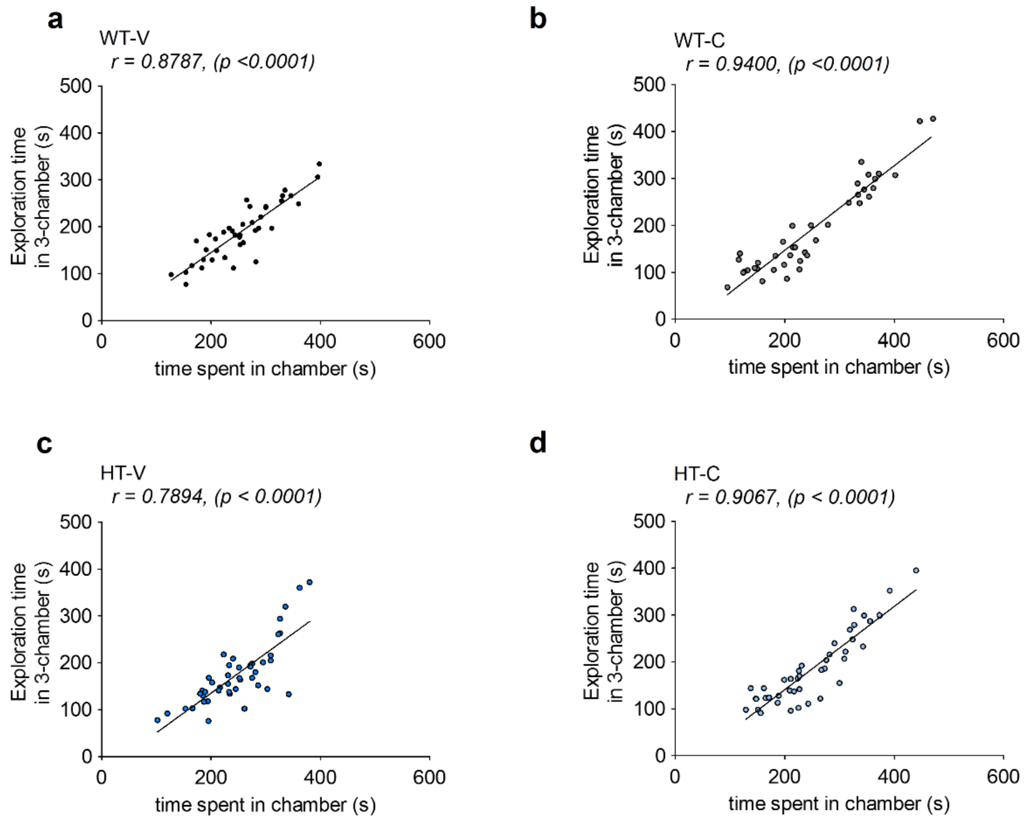

Supplementary Fig. 11. Strong correlation between chamber time and exploration time in *Tbr1*$^{+/-}$ (HT) mice.

(**a-d**) Time spent in chamber (chamber time) and time spent in exploring/sniffing the targets (O, S1, and S2) (exploration time) correlate well in *Tbr1*$^{+/-}$ mice at levels comparable to that in WT mice, as determined using the data from the three-chamber social interaction results from **Fig. 7** and **Supplementary Fig. 10** (Pearson correlation).

Suppl Fig 12

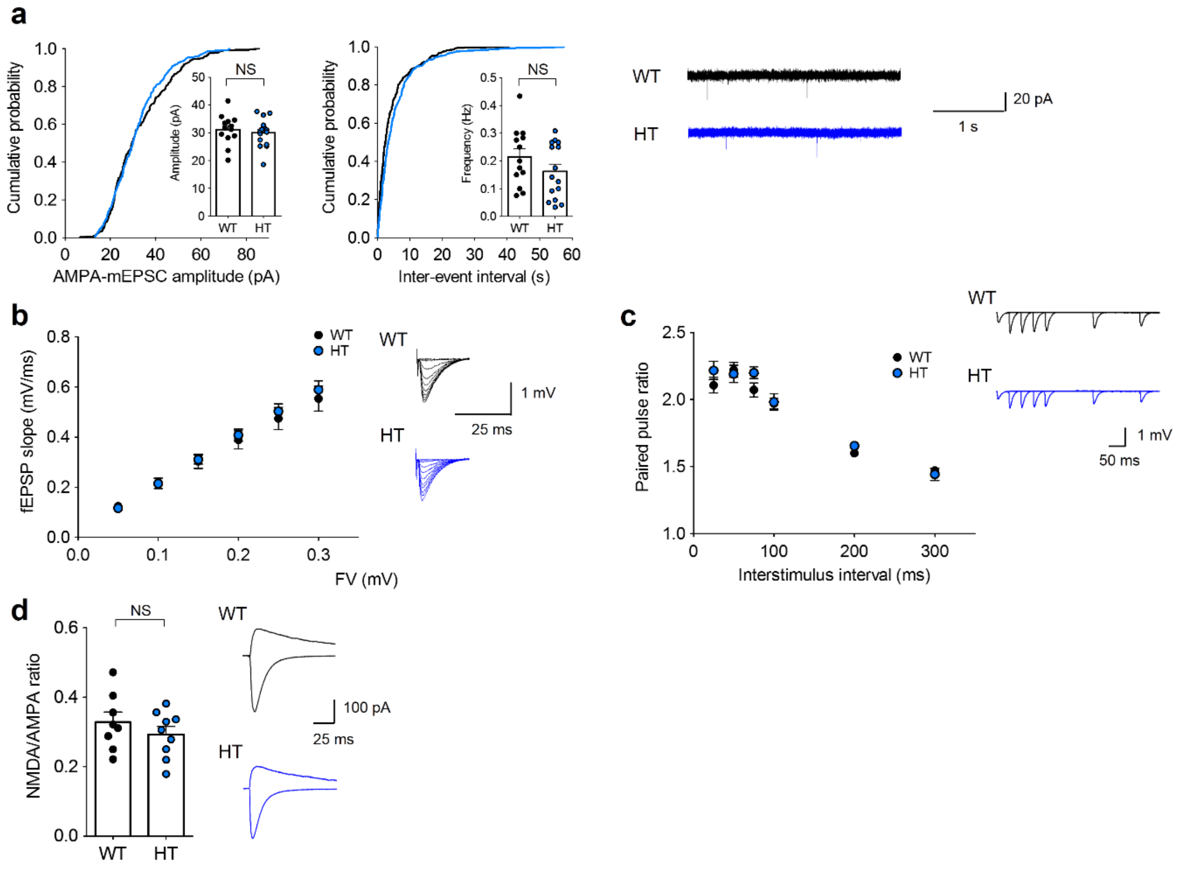

Supplementary Fig. 12. *Tbr1*$^{+/-}$ hippocampal SC-CA1 synapses show normal excitatory synaptic transmission

(**a**) *Tbr1*$^{+/-}$ hippocampal CA1 pyramidal neurons (3-5 weeks) show normal mEPSC amplitude and frequency. (n = 13 cells, 3 animals for WT, and 15 (3) for HT, NS, not significant, Student's t-test).

(**b**) *Tbr1*$^{+/-}$ hippocampal SC-CA1 synapses (3-5 weeks) show normal input-output ratio. (n = 10 slices, 3 animals for WT and HT; Student's t-test).

(**c**) *Tbr1*$^{+/-}$ hippocampal SC-CA1 synapses (3-5 weeks) show normal paired pulse ratio. (n = 10 slices, 3 animals for WT and HT; Student's t-test).

(**d**) *Tbr1*$^{+/-}$ hippocampal SC-CA1 synapses (3-5 weeks) show normal NMDA/AMPA ratio. (n = 8 cells (4 animals) for WT, 9 (5) for HT; Student's t-test). Data in all panels with error bars represent mean ± s.e.m.

Suppl Fig 13

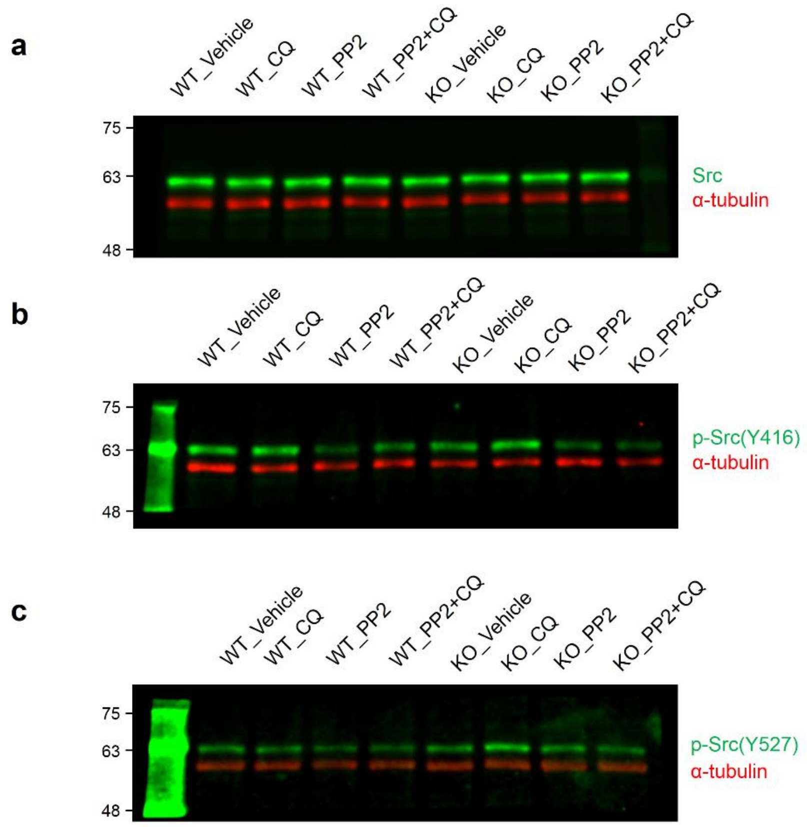

Supplementary Fig. 13. Full-size immunoblot images for Src phosphorylation in **Fig. 6a,d**.

(**a**) Total Src.

(**b**) Src phosphorylation at Y416.

(**c**) Src phosphorylation at Y527.
